# Role of an RNA pseudoknot involving the polyA tail in replication of *Pepino mosaic potexvirus* and related plant viruses

**DOI:** 10.1038/s41598-022-15598-5

**Published:** 2022-07-07

**Authors:** René C. L. Olsthoorn, Carolyn A. Owen, Ioannis C. Livieratos

**Affiliations:** 1 Leiden Institute of Chemistry, University of Leiden, P.O. Box 9502, 2300 RA Leiden, The Netherlands; 2 Department of Sustainable Agriculture, Mediterranean Agronomic Institute of Chania, Alsylio Agrokepion, 73100 Chania, Crete Greece

**Keywords:** Virology, RNA

## Abstract

*Pepino mosaic virus* (PepMV) is a potexvirus of the family *Alphaflexiviridae* within the order of *Tymovirales* that threatens tomato production worldwide. PepMV possesses a positive-strand RNA genome with a 5′-methylguanosine cap and a 3′-polyA tail. Previously, using partially-purified viral RNA polymerase important secondary structures within the 3′-untranslated region (UTR) of PepMV RNA were identified. Here we show that an RNA pseudoknot can be formed in the 3′-UTR that includes part of the polyA tail. Using protoplasts, we demonstrate that the pseudoknot is required for replication of PepMV RNA. Mutational analysis and native gel electrophoresis further show that the pseudoknot is stabilized by UAU base triples, as is the human telomerase RNA pseudoknot. The presence of a pseudoknot in several other members of the *Alpha-* and *Betaflexiviridae* is supported by covariance analysis and native gel electrophoresis of other potexvirus, capillovirus and trichovirus RNAs. The ubiquitous presence of the pseudoknot in viruses of the *Betaflexiviridae*, suggests that the pseudoknot is a typical trait of the *Betaflexiviridae* that may have been adopted by many potexviruses during evolution.

## Introduction

Plant viruses belonging to the order *Tymovirales* are currently subdivided into five families: *Alpha-*, *Beta-*, *Gamma-* and *Deltaflexiviridae*, and *Tymoviridae*^1^. All members possess a single plus-stranded RNA genome of 6–9 kilobases (kb) that encodes an RNA-dependent RNA polymerase (RdRP), a coat protein (CP), and one or more proteins involved in cell-to-cell movement of the virus. RdRP is translated from the genomic RNA, CP is translated from a subgenomic (sg) RNA, and the movement proteins are expressed from a gene that overlaps with the RdRP gene or from additional sgRNAs. Members of the *Tymoviridae* are distinct in that they form icosahedral-shaped particles and possess a tRNA-like structure (TLS) at the 3′-end of the RNA, which is usually charged with valine. *Alpha-*, *Beta-*, *Gamma-* and *Deltaflexiviridae* are characterized by flexuous, filamentous-shaped particles that are 470–1000 nm in length and 12–13 nm in diameter. Their genomes lack a TLS but have a 3′-polyA tail, which for *Bamboo mosaic potexvirus* (BaMV) has been shown to fold into a pseudoknot structure. So far, a pseudoknot structure has not been identified in other potexviruses or any other member of the *Alpha-*, *Beta-*, *Gamma-* and *Deltaflexiviridae*.

*Pepino mosaic virus* (PepMV) is a mechanically-transmitted potexvirus (*Alphaflexiviridae*) with an approximately 6.4 kb genome with a 5′-methylguanosine cap and a 3′-polyA tail^2^. The genome contains five open reading frames, coding for an RdRP, three proteins involved in virus movement, and CP, flanked by 5′- and 3′- untranslated regions (UTRs) of ~ 86 and ~ 64 nucleotides (nts), respectively^3^. In vivo and in vitro experiments have shown that three 3′-co-terminal sgRNAs are produced that express CP and the other proteins^4,5^.

**Figure 1:**
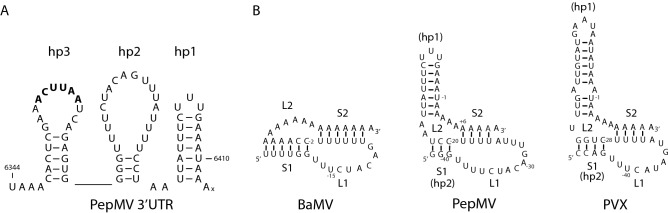
Secondary structure of PepMV 3′-UTR and representative pseudoknot structures of potexvirus RNAs. (**A**) Secondary structure of the 3′-UTR of PepMV isolate SP13. The stop codon of the CP ORF is boxed. The potex hexamer is indicated in boldface. The polyA tail starts after nt 6410. (**B**) BaMV pseudoknot and putative PepMV SP13 and PVX pseudoknots. The number of A residues in the tail is arbitrarily chosen. Please note: in contrast to the BaMV pseudoknot model of Tsai et al*.*^7^ we have positioned 6 As instead of 3 in L2 as the distance to bridge the minor groove of a 6-bp stem is ~ 30 Å whereas one nt generally bridges ~ 6 Å^11^, and in addition it may be assumed that a sixth A might interact with the first U in L1.

So far, secondary and tertiary structures have been identified in the 3′-UTRs of two other potexvirus genomes^6–8^. In the case of the type member *Potato virus X* (PVX) 3′-UTR, the RNA folds into three stem loops (hp1, hp2, hp3; numbered from the 3′-end^8^). hp1 is not required for minus-strand synthesis but hp2, which harbours a U-rich loop, is essential for plus– and minus-strand synthesis^8,9^. The BaMV 3′-UTR folds into a cloverleaf-like structure of four hairpins, followed by a pseudoknot that incorporates approximately 13 adenosines of the polyA tail^6,7^. The PepMV 3′-UTR folds into three stem-loop structures (Fig. 1A). In vitro assays using partially-purified viral polymerase have shown that hp1 is dispensable for minus-strand synthesis. hp3, which harbours the potexvirus hexamer, and hp2 are required for minus-strand synthesis. These two hairpins are very similar to their PVX counterparts, and accordingly, mutations in the U-rich loop of the PepMV hp2 were detrimental to minus-strand synthesis *in vitro*^10^.

Here we demonstrate that the U-rich motifs are involved in the formation of a pseudoknot that includes the polyA tail. Using protoplasts, we show that the pseudoknot is required for the replication of PepMV RNA. Mutational analysis and native gel electrophoresis show that the stabilization of the pseudoknot by UAU base triples is analogous to that of the human telomerase RNA pseudoknot. The presence of a telomerase-like pseudoknot in several other members of the *Flexiviridae* is supported by covariance analysis and by native gel electrophoresis of other potexvirus, capillovirus and trichovirus RNAs.

## Results

### Comparison of the 3′-UTRs of potexviruses shows potential pseudoknot formation involving the polyA tail

**Figure 2:**
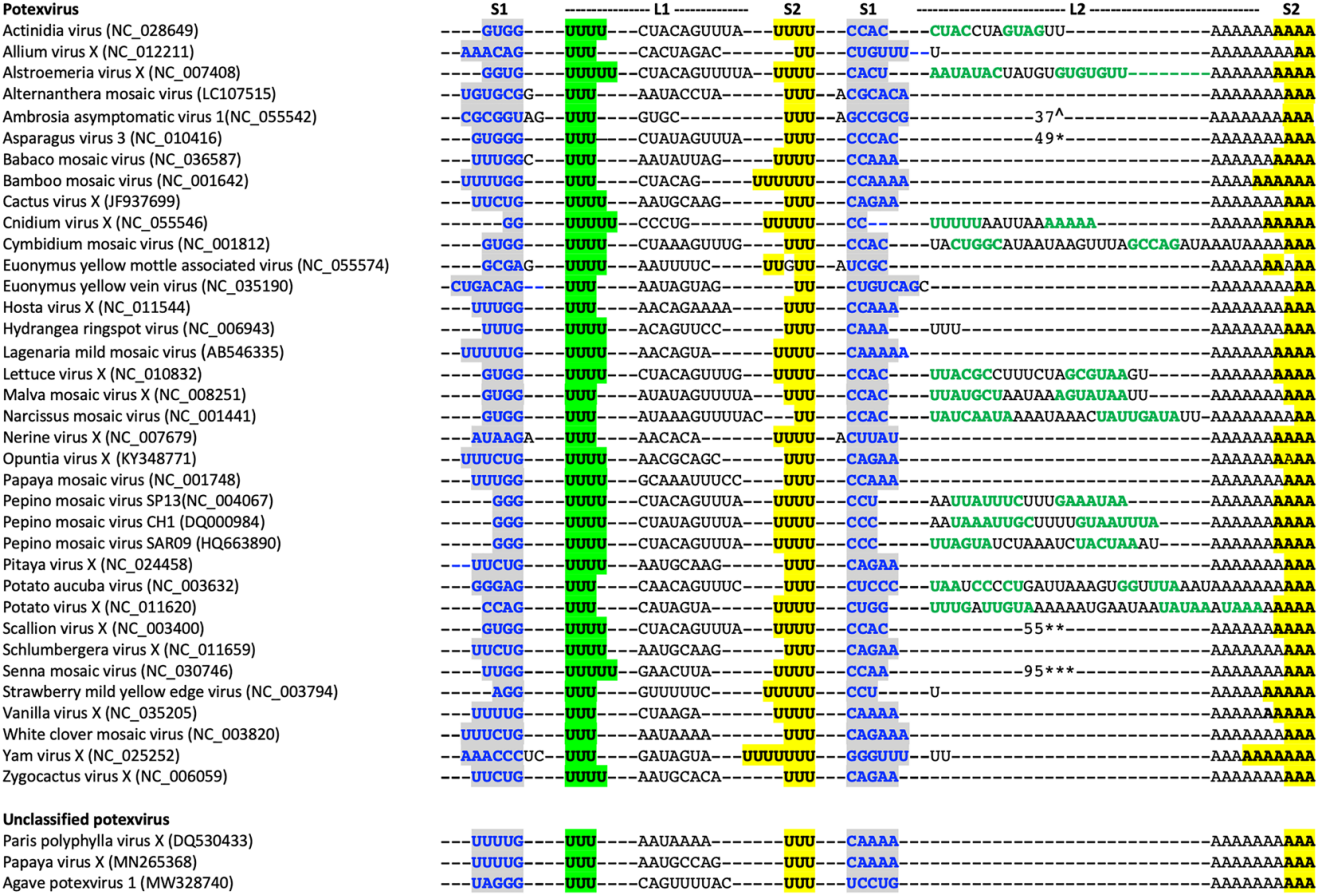
Alignment of 3′-ends of potexviruses capable of forming a pseudoknot structure. Bases in blue font form stem S1, those highlighted in yellow form stem S2. The 5′-proximal U-rich motif is highlighted in green. Putative hairpins formed by bases in L2 are shown in green font. *AGCAGACUAUCAUAUUUACUCUCUUUGAGCGUUAAUAAGUACGUGUGUU insert in L2 of *Asparagus virus 3.* **AUCAGACUCUCCAUCCUACUAGCUUUAUCCGCAUGUAUGAAUGUAAGUUUGUUUU insert in L2 of *Scallion virus X.* ***UUCUUGCCACCGCCAGAGUGAGAGUCUAGUUUAGUCAGCCCGUUGUUUUCGCACUUUUGUUGGGGCUAUUGAGUUUUCAAAAGUGCUGUCUAGCU insert in L2 of *Senna mosaic virus.*

Previously, the 3′-UTR of BaMV RNA was shown to terminate in a pseudoknot involving approximately 13 adenosines of the polyA tail (Fig. 1B^7^) but an analogous structure had not been detected nor proposed for any related potexviruses. Alignment of potexvirus RNA sequences, however, shows that a stem equivalent to stem S1 of the BaMV pseudoknot can be formed in the majority of potexviruses, including three unclassified potexviruses (Fig. 2, blue characters); while equivalent sequences could not be detected in a further 13 potexvirus species. The existence of S1 is further supported by the presence of covariations; almost every possible base-pair can be found in S1, indicating that base-pairing potential of bases at these positions in the genome is preserved among these viruses. An exception may be the strongly conserved GC pair closest to loop L1; in only a few potexviruses is this pair CG, AU, GU, or UG. In approximately half of these potexvirus RNAs, adenosines from the polyA tail are involved in the formation of S1, while in the remainder the polyA tail is not involved in S1 and is preceded by 14–95 nts with the ability to form an AU rich stem-loop structure (indicated in green font).

By analogy with the BaMV pseudoknot, all sequences contain U-rich motifs that flank the nucleotides of stem 1 (highlighted in green and yellow). For BaMV it was shown that the 3′-proximal Us (highlighted in yellow) can base-pair with 6 As of the polyA tail (highlighted in yellow), thereby forming stem S2. In the potexvirus RNAs shown here, 2–7 Us are available for base-pairing with As from the polyA tail. The conserved 5′-proximal U-rich motif (highlighted in green) can in principle also base-pair with As from the polyA tail but this would lead to an unusual and rare pseudoknot structure wherein the polyA tail has to span both S1 and S2. Since we found that the 5′-proximal Us are involved in base triples (see below) we did not consider this alternative pseudoknot conformation.

Potexvirus pseudoknots can thus be grouped into those that have additional sequences capable of forming a stem-loop structure within the loop L2 of the pseudoknot (*e.g.* PepMV and PVX), and those for which L2 is composed of As from the polyA tail (*e.g.* BaMV). A few viruses have one (*Allium virus X* and *Strawberry mild yellow edge virus*), two (*Yam virus X*) or three Us (*Hydrangea ringspot virus*) that separate S1 from the polyA tail. While the role of this additional structure remains unknown and may be host-related, its presence is probably not required for the synthesis of minus- or plus-strand RNA, as was reported for PVX^8^. Also, replication of PepMV RNA in vitro and in protoplasts is not affected by removal of this hairpin^10^.

### Effect of polyA-length on replication in vivo

The length of the polyA tail is obviously crucial for the formation of the pseudoknot and indeed BaMV transcripts with 10 or less As are not infectious as they probably do not allow formation of the pseudoknot^7^. To investigate the length of the polyA tail required for replication of PepMV, a full-length PepMV SP13 cDNA clone was used as template for PCR mutagenesis and in vitro transcription. In the final capped mRNAs U_−18_ has been substituted by a C, a natural variation found in several other PepMV isolates (Fig. 2). This substitution is thought to stabilize stem 1 of the pseudoknot^10^.

**Figure 3:**
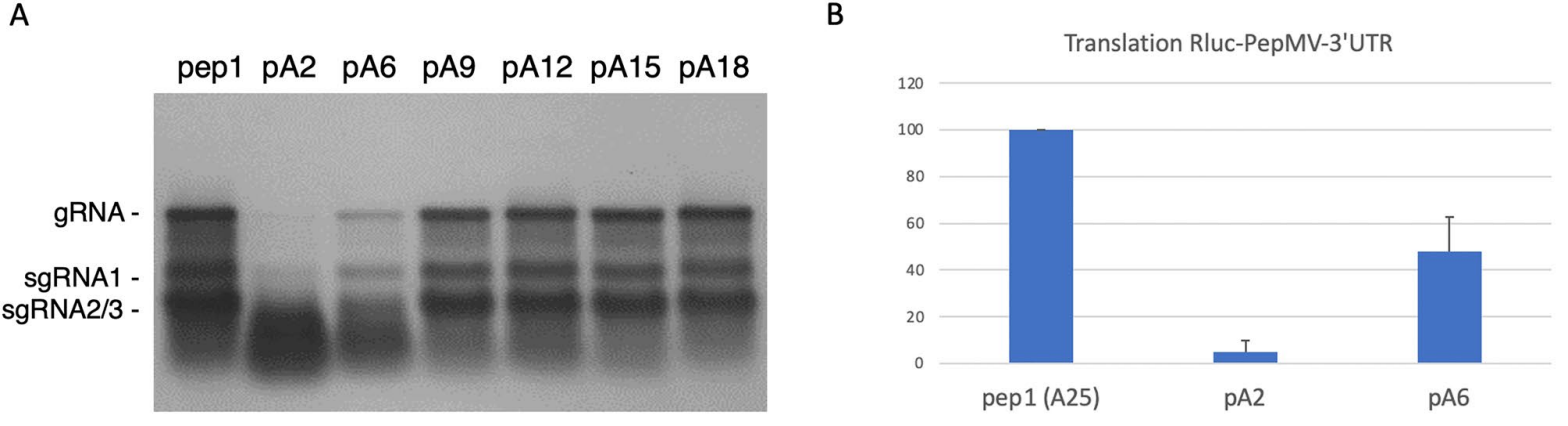
The effect of polyA tail length on the replication of PepMV. (**A**) Northern blot showing the accumulation of PepMV gRNA and sgRNAs in *N. benthamiana* protoplasts. pep1: wt PepMV with A25, A2, A6, A9, A12, A15, A18. The migration of gRNA and sgRNAs is indicated. We note that sgRNA2 and 3 comigrate on these gels. (**B**) Effect of polyA tail length on translation efficiency of the PepMV-3′UTR in protoplasts. Error bars indicate standard deviation of 2 experiments.

Transfection of *Nicotiana benthamiana* mesophyll protoplasts with PepMV RNA with a tail of only 2 As (pA2) led to barely detectable accumulation of gRNA and sgRNAs (Fig. 3A). However, the presence of 6 As (pA6) resulted in significantly enhanced replication while with 9 As (pA9) the replication levels were close to those of the reference construct incorporating 25 As (pep1). The presence of 12, 15, or 18 As did not result in a detectable further increase in replication. The construct with 25 As (pep1) will be referred to as wild type (wt) henceforth.

As the polyA tail is also necessary for translation, an essential early process in PepMV infection, it is likely that the number of As will also affect translation. In an attempt to differentiate the effects of polyA tail-length on replication and translation, templates were made in which a *Renilla* luciferase gene was fused to the PepMV 3′ UTR that incorporated a polyA tail of 2, 6, or 25 As. Capped RNA transcripts synthesized from these templates were transfected into *N. benthamiana* protoplasts and luciferase activity as a measure of protein translation was determined 16 h post-transfection. The presence of 2 As resulted in very low levels of translation (~ 5%), whereas translation of the transcript incorporating 6 As was only twofold less than the wt RNA with 25 As (Fig. 3B). This result suggests that the low replication observed with a polyA tail of 6 nts is mainly attributable to a decrease in minus-strand RNA synthesis, rather than to diminished translation.

### Stem S1 requirements

**Figure 4:**
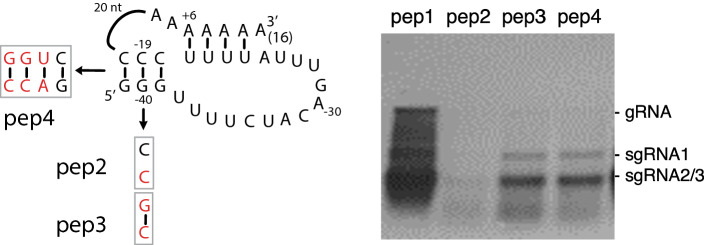
Role of S1 length and stability in replication. For the sake of clarity hp1 is not depicted. Northern blot showing the accumulation of PepMV gRNA and sgRNAs in *N. benthamiana* protoplasts.

Previously, we demonstrated that base pairing in hp1 was important for the replication of PepMV RNA in vitro. Here we investigated whether the integrity of stem S1 (*i.e.* the stem of hp2) is important for replication in vivo. Disruption of the middle GC bp to a CC mismatch (pep2) greatly reduced replication. (Fig. 4). Restoring S1 by converting the CC mismatch to a CG bp (pep3) improved replication to some extent but the level was substantially lower than for the wt. Replacing S1 with the corresponding stem of PVX (pep4) also resulted in similarly reduced replication. This is potentially due to the altered stability of S1: pep3 − 5.80 kcal/mol, pep4 − 7.50 kcal/mol, wt − 6.60 kcal/mol (calculated by Mfold^12^), although a loss or change of putative tertiary interactions could also contribute to a lower accumulation. Note that a CG pair as in pep3 is rarely found as the penultimate bp in stem 1 of potexviruses (Fig. 2).

### Role of putative UAU base triples in replication

**Figure 5:**
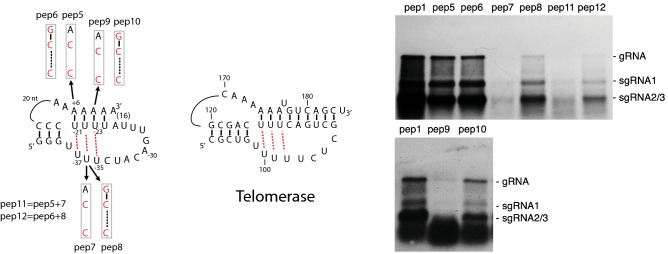
Putative UAU triples in PepMV by analogy to the human telomerase pseudoknot. Northern blots showing the accumulation of PepMV gRNA and sgRNAs in *N. benthamiana* protoplasts after transfection with the indicated constructs.

Pseudoknots are stabilized by various factors, including interactions between bases in the loops and base pairs in the stems^13^. For example, UAU base triples formed between U residues in the loop and AU bps in the stem have been shown to be essential for the structure and stability of the human telomerase pseudoknot (Fig. 5^14^) and of other noncoding RNAs^15^. In these studies, UAU base triples could be functionally replaced with isosteric CGC triples. To investigate the presence of UAU base triples in PepMV we disrupted three potential UAU triples identified from homology with the human telomerase pseudoknot, by replacing Us with Cs (pep5, pep7, pep9). Replication of pep5 was moderately reduced, while replication of pep7 and pep9 was severely reduced (Fig. 5). By introducing an A-to-G change in the polyA tail of these mutants we attempted to restore base-pairing in stem S2 of the pseudoknot and generate an isomorphic CGC base triple (Fig. 5; pep6, pep8, pep10). This introduction did not result in increased accumulation of PepMV RNAs for pep6 compared to pep5, but did lead to a substantial increase in accumulation of PepMV RNAs for pep8 and pep10 relative to their respective CAC mutants pep7 and pep9 (Fig. 5). The simultaneous mutation of the triples A_+6_U_−21_U_−37_ and A_+7_U_−22_U_−36_ yielded results similar to mutation of A_+7_U_−22_U_−36_ alone (Fig. 5, compare pep11 and pep7) while introduction of CGC triples at these positions again partially restored replication (Fig. 5, pep12). These data strongly suggest that UAU triples are formed between A_+6_U_−21_U_−37_, A_+7_U_−22_U_−36_ and A_+8_U_−23_U_−35_, in a structure analogous to the human telomerase pseudoknot, but that the A_+6_U_−21_U_−37_ triple is quite tolerant of mutation.

### Native gel electrophoresis of model potexvirus pseudoknots

**Figure 6:**
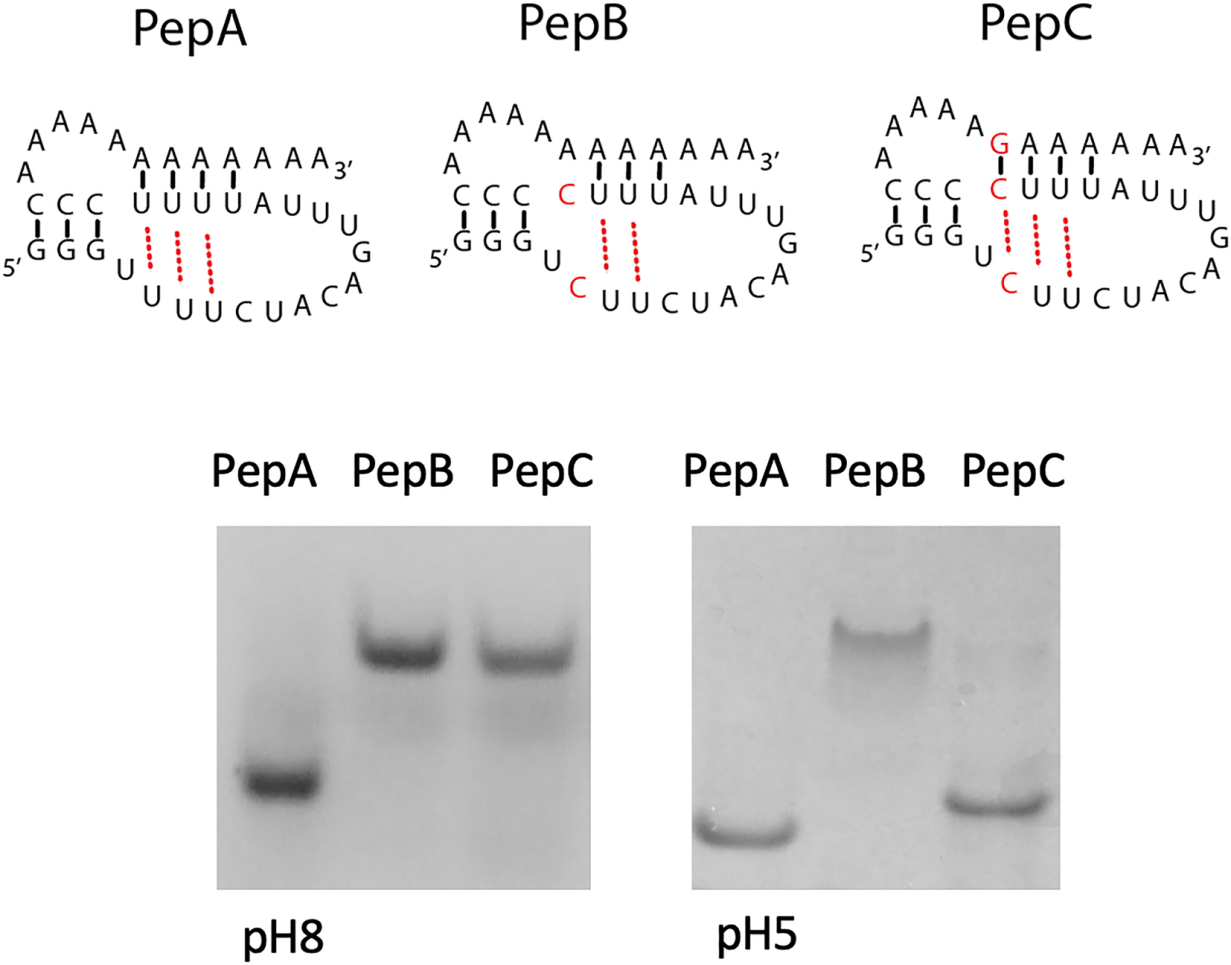
Native gel electrophoresis at pH 8 and 5 of PepMV pseudoknot RNAs with wild type (PepA) or mutant base triples (PepB, PepC). RNAs were visualized by Stains-All.

To study the importance of base triples for the structure of PepMV and other potexvirus pseudoknots, we used short RNA oligonucleotides and native gel electrophoresis. In PepA (36 nts) which corresponds to the PepMV pseudoknot from which hp1 has been removed, potentially three UAU base triples can be formed, whereas in PepB two U-to-C changes disrupt one base triple, and in PepC, in addition to these two U-to-C changes, an A-to-G change in the polyA tail is introduced to create a CGC base triple. Figure 6 shows that PepA migrated faster than both PepB and PepC, indicating a more compact structure. Interestingly, at pH5 when cytosines become protonated, the migration of PepC equaled that of PepA, while PepB still lagged behind. Since the only difference between PepB and PepC is the substitution of one G for an A in the tail, the most likely explanation for their difference must be the formation of a CGC triple. 1D proton NMR spectroscopy of PepA also supported the existence of a pseudoknot structure for this RNA (Supplementary Fig. S1). We note that CGC triples are naturally found in a variety of RNAs and can substitute for UAU triples but that in vitro protonation of one of the cytosines is generally required for their formation.

**Figure 7:**
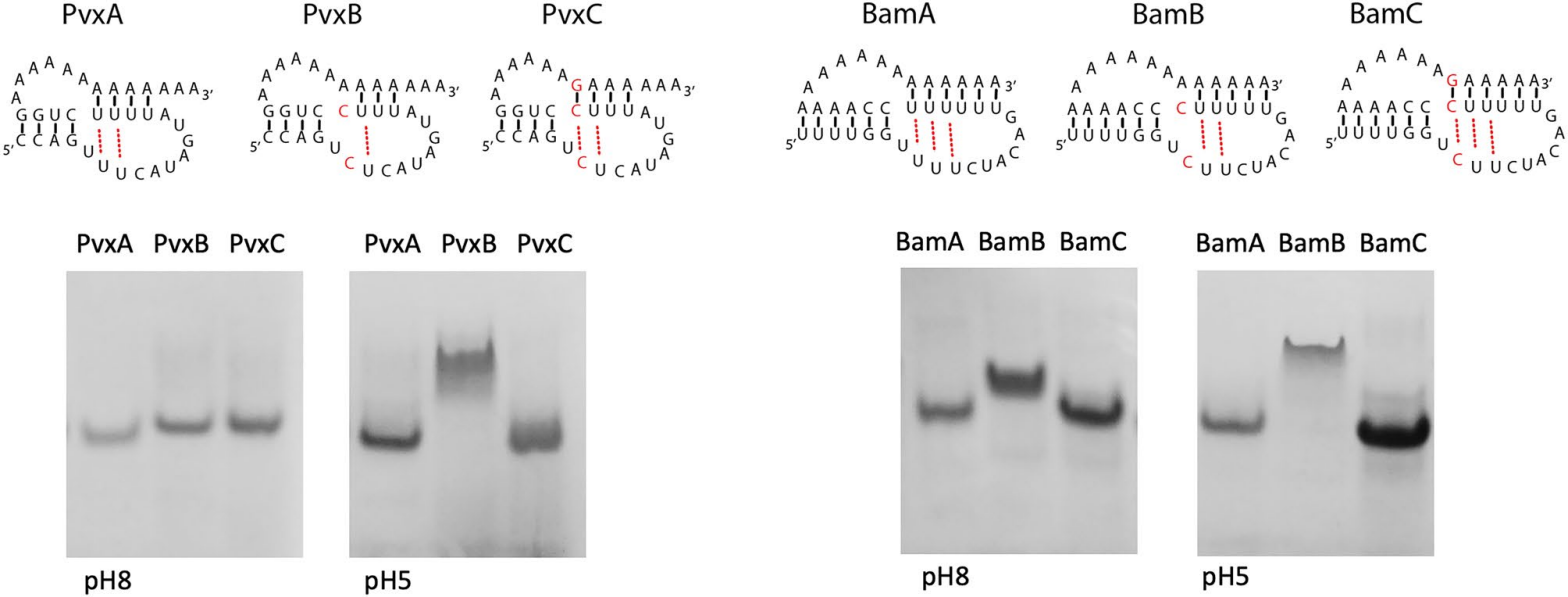
Native gel electrophoresis at pH 8 and 5 of PVX and BaMV pseudoknot RNAs. RNAs were visualized by Stains-All.

Similar results were obtained with PvxA, using a model oligonucleotide of 34 nts corresponding to the 3′-end of PVX RNA but lacking the terminal hairpin that is not required for minus-strand synthesis^8^. At pH 8 PvxA migrated faster than PvxB which has two U to C changes that disrupt the UAU triple, and PvxC with the CGC triple (Fig. 7). Again, at low pH PvxC co-migrated with PvxA while PvxB did not. The BaMV pseudoknot (BamA) migrated faster than the double mutant (BamB) demonstrating that base triples are also present in BaMV RNA. Interestingly, BamC with the CGC triple migrated as fast as BamA at both high and low pH, suggesting that in the context of the BaMV sequence the CGC triple is stable at pH 8. In conclusion, PVX and BaMV RNAs also adopt a pseudoknot structure that is stabilized by UAU base triples.

### Pseudoknot conservation in other Alpha- and Betaflexiviridae

In order to establish whether the 3′-UTRs of other *Alpha-* and *Betaflexiviridae* could adopt a similar pseudoknot, an alignment was made of all available sequences from the *Alphaviridae*. This alignment indicated that pseudoknots may exist in the genus *Mandarivirus*, but not in *Allexi-*, *Botrex-*, *Lola-*, *Platypu-* and *Sclerodarnaviruses* (see full alignment supplementary Fig. S2). Among the *Betaflexiviridae*, the pseudoknot was found within the subfamily *Quinvirinae* in the genera *Carlavirus*, *Foveavirus*, and *Robigovirus*, as well as in some unclassified species. Within the subfamily *Trivirinae* similar pseudoknots could be found in all genera except for *Vitivirus*. In all but 4 viruses the pseudoknot stems S1 and S2 consists of at least three base pairs and the majority have the potential to form three UAU triples. In contrast to those of the potexviruses a large number of these pseudoknots possess a single C or A residue between stems S1 and S2. This would not necessarily prevent pseudoknot formation as the existence of unpaired bases at the junction between two coaxially stacked stems of a pseudoknot is not uncommon^16^. A few sequences, mostly from trichoviruses, feature unpaired bases separating the S1 and the U-stretch involved in UAU base triples.

**Figure 8:**
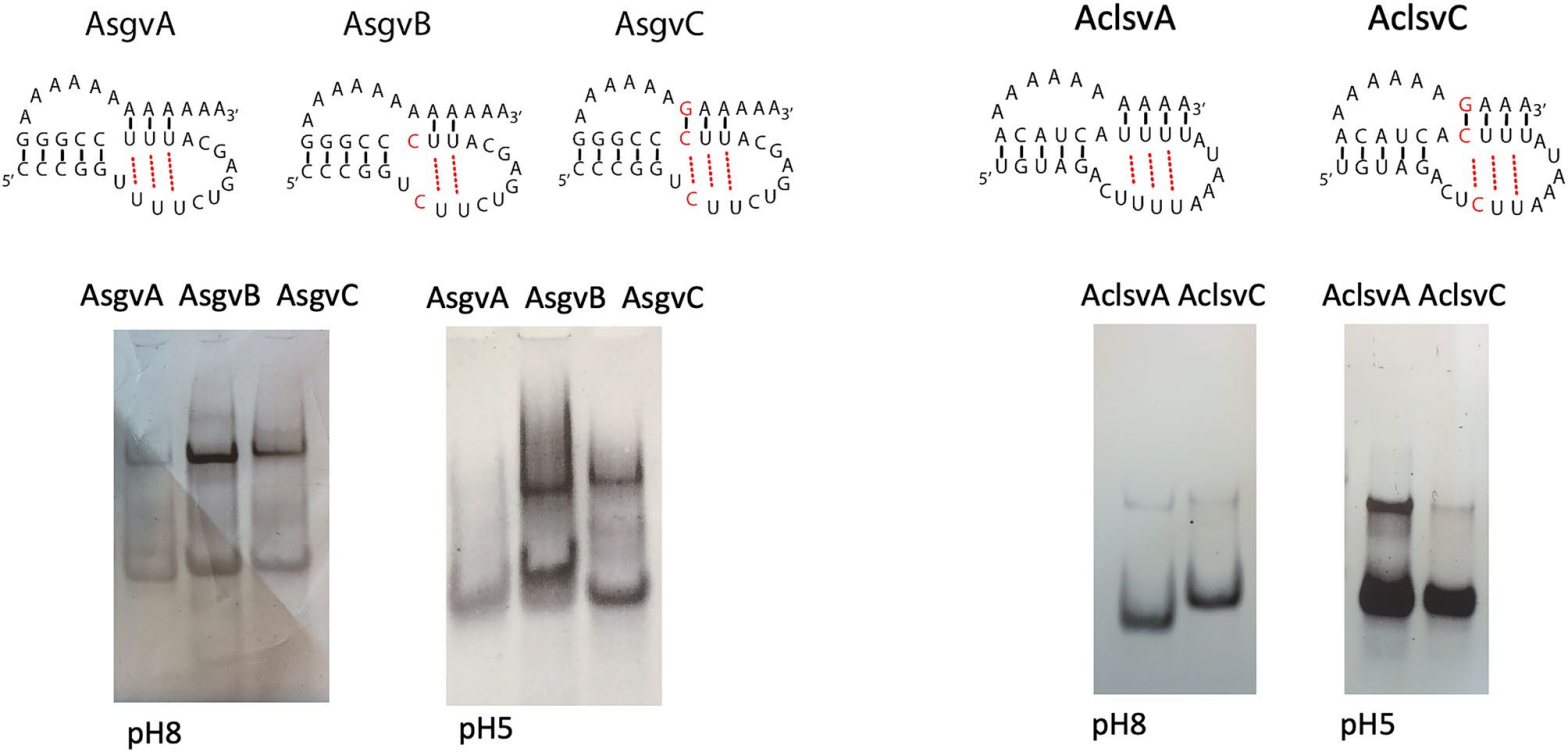
Native gel electrophoresis at pH 8 and 5 of *Apple stem grooving capillovirus* (AsgvA, B, C) and *Apple chlorotic leaf spot trichovirus* (AclsvA and C) pseudoknot RNAs. RNAs were visualized by EtBr staining.

We tested whether pseudoknot formation is possible for the capillovirus *Apple stem grooving virus* (AsgvA), and the trichovirus *Apple chlorotic leaf spot virus* (AclsvA) which has an A at the junction between the stems and 2 nts upstream of the U-stretch (Fig. 8). Both wt RNAs, AsgvA and AclsvA, migrated faster through high pH gels than mutants in which the UAU triple was replaced by a CGC (AsgvC, AclsvC) or a CAC mismatch (AsgvB). At low pH, migration of wt and the CGC mutants was identical, indicating that the protonated CGC triple restored the pseudoknot, whereas the CAC mutant (AsgvB) migrated even more slowly. 1D proton NMR spectroscopy of AsgvA and AsgvB samples also showed clear differences in the area of UA base pairs, indicative of pseudoknot formation in AsgvA (Supplementary Fig. S3). These results show that a pseudoknot can be formed in capillo- and trichoviruses and that intervening nucleotides between stems do not interfere with pseudoknot formation.

## Discussion

To date, the only member of the *Potexvirus* genu*s* shown to possess a 3′-terminal pseudoknot was BaMV. However, the data in this work strongly suggest the presence of a pseudoknot structure involving the polyA tail in the RNA of PepMV, and also in the majority of the potexviruse*s*. The PepMV pseudoknot is very likely essential for replication of the RNA as translation was less sensitive to the length of the polyA tail. With a 6 A-tail translation was only twofold lower than wt (25 As), whereas replication was strongly reduced. On the other hand, 9 As were sufficient to obtain wt levels of RNA replication in vivo. This contrasts with the results of previous in vitro experiments using partially purified PepMV replicase that showed no detectable minus strand synthesis with polyA tails of 10 or 15 As^5^. This discrepancy may be due to the addition of As in vivo by a cellular polymerase, acting in competition with the degradation of templates with short polyA tails. Folding of the 3′- terminus into a pseudoknot with 6, 10 or 15 As may protect against this degradation in vivo and allow further extension of the polyA tail to produce a replicable template.

For BaMV, no replication in vivo was detected with a polyA tail of 10 A or less. Since in BaMV 4 As are necessary for the formation of stem S1 of the pseudoknot, the remaining 6 As are apparently not sufficient to make up stem S2 and loop L2. The minimum length of the polyA tail for replication of another well-studied potexvirus, PVX has not been definitively determined: 8 As were reported to yield very low replication but the transcript also incorporated 4 additional nts derived from a restriction site^17,18^. Pillai-Nair et al*.*^8^ achieved successful infection of protoplasts using PVX transcripts with 17 As that also contained the 4 additional nts. In any case, as 3 As form part of hp1, the remaining 14 As are sufficient to form the proposed pseudoknot for PVX.

Mutational analysis and native gel electrophoresis also indicated that the PepMV pseudoknot is stabilized by a number of UAU base triples, as their disruption reduced replication whereas their substitution with isosteric CGC base triples preserved replication. We observed that not all triples are equally important. The triple closest to stem S1 can be disrupted by CAC (pep5) or replaced by CGC (pep6) without much effect on replication. On native gels, among PepMV RNA oligonucleotides lacking hp1, mutation of the triple closest to S1 was sufficient to change the migration and structure of the pseudoknot, and alter the NMR spectrum. Whether hp1 exerts a stabilizing effect on the pseudoknot and possibly mitigates the effect of destabilizing mutations remains to be investigated.

CGC triples did not, however, fully restore replication. One reason could be that CGC triples require protonation of one of the cytosines which at neutral pH occurs slowly but can be accelerated by neighbouring UAU triples^19^. Other possible reasons for the lower levels of replication are: (i) the PepMV triples are in a different register and so were here inadvertently introduced at the wrong position, thereby forcing the structure into a conformation less favorable for replication. At present, we cannot rule out this possibility, but the fact that constructs with CGC triples replicated better than their corresponding CAC mismatches at least indicates that the PepMV pseudoknot is stabilized by UAU triples. Elucidation of the 3D structure of the PepMV pseudoknot will help to determine the exact nature of these triples and also the extent of the homology with the human telomerase pseudoknot, or with other pseudoknots stabilized by UAU triples^20^. (ii) The interruption of the polyA tail by one or more G may adversely affect translation leading to decrease in viral protein levels and, indirectly, to less synthesis of gRNA and sgRNAs. Currently, no data exist to support this hypothesis. (iv) The CGC triple may interfere with the formation of an alternate hairpin structure which in the wt is stabilized by UU base pairs, but in the mutants these are replaced by CC or CU pairs. This hairpin structure may be the binding site for a host factor such as that shown by Sriskanda et al*.* to bind to the U-rich sequence in L1 of PVX^9^, although it cannot be excluded that this factor recognizes the UAU triples, as mutation of these Us would also disrupt this interaction.

Based on the structural resemblance to the PepMV pseudoknot, and data from native gel electrophoresis, we also propose a 3′-terminal pseudoknot involving the polyA tail for PVX. Although previous structure-probing data on the PVX 3′-UTR did not show clear evidence of the U-rich motifs being involved in base pairing, Pillai-Nair et al*.*^8^ found U_−40_, U_−41_ and U_−42_, that we predict to be involved in the formation of the UAU base triples, to be largely protected from modification by CMCT. The 4 Us involved in the proposed AU bps in stem S2, however, were more accessible to CMCT but still less so than other Us in hairpin loops in the 3′-UTR of PVX RNA. Interestingly, U_−15_ to U_−17_ in loop L1 of the BaMV pseudoknot are protected from RNAse A cleavage but are a substrate for the dsRNA specific RNAse V1^9^ which supports the presence of UAU triples in the BaMV pseudoknot as well. Other data that support the presence of a pseudoknot in PVX come from a study using BAMV-PVX hybrids^21^ showing that the PVX 3-’UTR can partially substitute for the potex hexamer and pseudoknot domains of BaMV in in vitro minus-strand synthesis. Future research should demonstrate the requirement of the pseudoknot for the replication of PVX.

The pseudoknot can be found in 34 classified and three unclassified potexviruses but is absent in 13 classified potexviruses. These 13 viruses cluster together in five subclades based on similarities in their replicase and coat protein genes^22^ which we have tentatively assigned here to group 1 *Plantago asiatica mosaic virus*, *Tulip virus X* and *Cassava common mosaic virus* [CasCMV]); group 2 *Lily virus X*, *Phaius virus X* and *Mint virus X* ; group 3 (*Clover yellow mosaic virus* [ClYMV] and *Tamus red mosaic virus* [TaRMV]); group 4 (*Foxtail mosaic virus* [FxMV] and *Turtle grass virus X* [TGVX]); group 5 (*Cassava virus X*, *Cassava mosaic virus X* [CasMV], and *Cassava Colombian symptomless virus*. Careful inspection of their 3′-ends shows that group 1 shares a stem-loop structure with certain marafiviruses of the *Tymoviridae* (Fig. S4). The 3′-ends of group twofold into a so-called pseudotriloop hairpin which functions as a recognition element for *Brome mosaic virus* replicase^23^ but is also found at the 3′-ends of some allexiviruses and the botrexvirus *Botrytis virus X* (Fig. S4). Presumably, during evolution these potexviruses exchanged the 3′ pseudoknot with the 3′ stem-loop of plant viruses from a nearby family. Such events have also been documented for other plant viruses^24,25^ and avian caliciviruses^26^.

ClYMV and TaRMV (Group 3) both form a stem-loop structure with a similar loop sequence that is not found in other viruses (Fig. S4). Cassava viruses (group 5) also form a stem-loop structure with loop sequences that, apart from a CAG sequence, have little in common. Interestingly, the loop sequence of CasMV (ACAGUUUA) is very similar to loop L1 of the pseudoknot of other potexviruses like PepMV, *Actinidia virus*, *Alstroemeria virus X*, etc. Group 4 viruses FxMV and TGVX form a hairpin of 6–7 bp with a loop sequence AAUGCACA that shows strong homology with the loop sequence of the pseudoknot of *Zygocactus virus X*, *Cactus virus X*, *Schlumbergera virus X*, *Pitaya virus* (Fig. 2). This is remarkable as FxMV and TGVX are more closely related to BaMV, which has the CUACAG motif that is shared by potexviruses like PepMV. The exact function of this motif is not known, but the fact that satellite RNAs of BaMV^27^ which are replicated by the same RdRP do not possess this motif suggest that it is bound by a host factor rather than by the RdRP itself. A potential host factor may be NbHsp90 that was shown to bind to the BaMV pseudoknot but not to the 3′end of its satellite RNA that lacks the pseudoknot^28^.

This type of pseudoknot involving the polyA tail can be found in one other genus, *Mandarivirus*, of the *Alphaflexiviridae*, but not in the genera *Allexivirus*, *Botrexvirus*, *Lolavirus*, *Platypuvirus*, and *Sclerodarnavirus* of the *Alphaflexiviridae*. The only allexivirus sequence that was found to harbour a pseudoknot is the unclassified allexivirus *Garlic yellow virus*, but this virus is closely related to *Allium carlavirus A*, an unclassified carlavirus. In the related *Betaflexiviridae* however, the pseudoknot is widespread: it is found in 11 genera, and is only not present in the genus *Vitivirus*. The 3′-ends of vitivirus RNAs can be folded into a hairpin that in many cases is stabilized by base pairs involving the polyA tail (Fig. S4) similar to allexiviruses. Thus the pseudoknot seems to be a typical trait of the *Betaflexiviridae* that was somehow adopted by many potexviruses during evolution. It will be of considerable interest to find out whether the pseudoknot also plays an important role in the replication of viruses belonging to the *Betaflexiviridae*.

## Methods

### Synthesis of RNA templates for protoplast inoculation

DNA fragments representing differently-sized sequences of the PepMV plus-strand genome were amplified from the pTOPO-T7 PepXL6 DNA template. All PCRs were carried out using LA Taq DNA polymerase (TAKARA), the template DNA was restricted with *Dpn*I prior to purification of the generated products by agarose gel electrophoresis and used as templates for in vitro transcription using the mMESSAGE mMACHINE T7 RNA kit (Ambion). The 5′-methylguanosine capped RNA transcripts produced were purified as described in the Ambion manual, and assayed by spectrophotometer before use in downstream experiments.

### PepMV replication assays

Full length PepMV cDNA templates were amplified using a T7pep forward oligonucleotide primer and one of a set of 3′-co-terminal reverse oligonucleotide primers (Supplementary Table S1), purified and transcribed. Five µg of each transcript being used to inoculate 5 × 10^5^ *N. benthamiana* mesophyll protoplasts in the presence of PEG 4000, before incubation for 24 h at 25 °C under constant light. Protoplasts were harvested and total RNA was isolated using Trizol reagent (Invitrogen) according to the manufacturer’s instructions. For subsequent northern blot analysis, 5 µg of each total RNA was resolved, transferred to charged nylon membranes and probed with a Dig-labelled (-) single stranded RNA probe corresponding to the PepMV CP gene coding sequence.

### Translation assays

The RLuc-PepMV-3UTR plasmid was obtained by insertion of a DNA fragment covering the PepMV 3′UTR (Baseclear, Leiden, The Netherlands) into a *Renilla* luciferase reporter plasmid previously described^29^. Templates for transcription were obtained by PCR using the forward primer SP6FLU and reverse primer (pep1, pA2, or pA6—Supplementary Table S1). Transcription reactions were carried out as described above, but with the kit enzyme mix substituted with a 7:2:1 mixture of SP6 polymerase, recombinant RNAse inhibitor, and inorganic pyrophosphatase (all New England Biolabs). 5 × 10^5^ *N. benthamiana* protoplasts were transfected with 3 µg of RNA and after 16 h incubation at 25 °C under constant light, were freeze-dried and sent by airmail for analysis at Leiden University. Upon arrival material was resuspended in 100ul Tris (10 mM pH 8) and luciferase activity in 50 µl samples was measured using a GloMax multi system (Promega).

### Native PAGE

RNA oligonucleotides were purchased from Merck (Sigma-Aldrich) at 0.05 nmole scale/desalted. 100–200 pmol of each RNA were loaded onto polyacrylamide gels containing 12% or 16% acrylamide:bisacrylamide (19:1), Tris (40 mM), acetate (20 mM), EDTA (1 mM) pH 8.3, with 1.5 mM MgAc_2_. Gels were run in TAEM buffer at ~ 85 V and 12 mA for ~ 4 h in a cold room. For acidic PAGE the pH was adjusted by the addition of acetic acid to all buffers. RNA was visualized using EtBr or Stains-All (Sigma Aldrich).

### NMR spectroscopy

^1^H NMR experiments were recorded at 278° Kelvin on a Bruker 600 MHz spectrometer equipped with a cryoprobe using Watergate suppression. RNA oligonucleotides were dissolved in 10 mM Na_2_HPO_4_/NaH_2_PO_4_ buffer (pH 6.7), 0.5 mM MgSO_4_ and 10% D_2_O to a final concentration of ~ 0.25 mM.

## Supplementary Information

Supplementary Information.

## Data Availability

The datasets generated during the current study are available from the corresponding author on reasonable request.
