# Supplementary material for: Role of an RNA pseudoknot involving the polyA tail in replication of Pepino mosaic potexvirus and related plant viruses
Source: Sci Rep. 2022 Jul 7;12:11532. doi: 10.1038/s41598-022-15598-5 (PMC9262919; doi:10.1038/s41598-022-15598-5)
Supplement: Supplementary file 1 — Supplementary Information. [file 41598_2022_15598_MOESM1_ESM.pdf]

Supplementary material for:

Role of an RNA pseudoknot involving the polyA tail in replication of Pepino mosaic potexvirus and related plant viruses

René C. L. Olsthoorn[1]*, Carolyn A. Owen[2], and Ioannis C. Livieratos[2]

[1]*Leiden institute of Chemistry, University of Leiden, PO Box 9502, 2300 RA Leiden, The Netherlands*
[2]*Department of Sustainable Agriculture, Mediterranean Agronomic Institute of Chania, Alsylio Agrokepion, GR-73100, Chania, Crete, Greece*

*Corresponding. olsthoor@chem.leidenuniv.nl

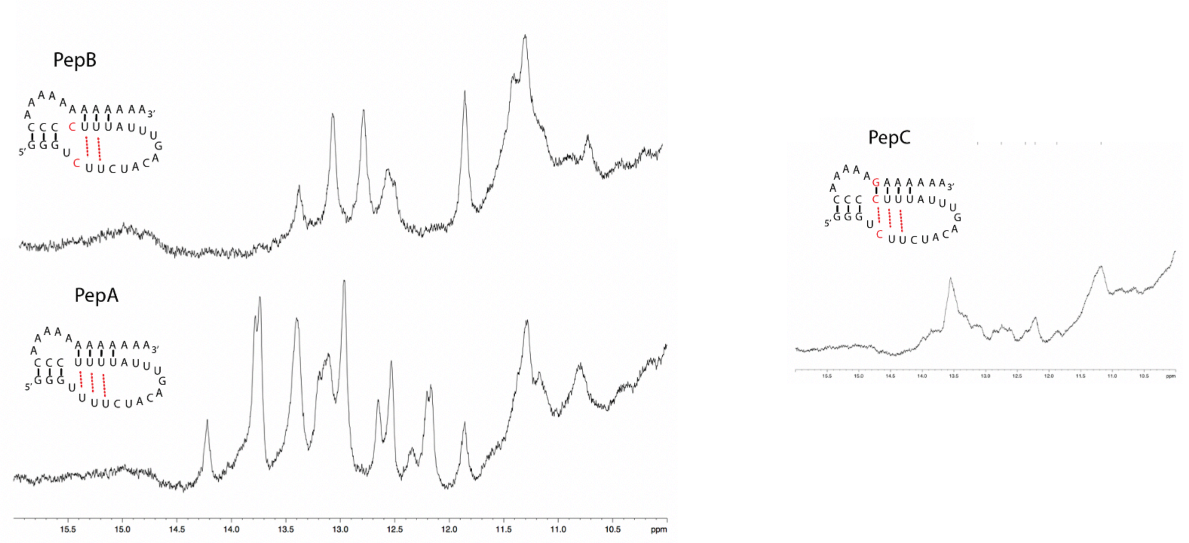

Fig. S1. 1D proton NMR of PepA, PepB and PepC. The three peaks downfield of 13.5 ppm in the spectrum of PepA are tentatively assigned to three AU base pairs of stem 2. These resonances are absent in the spectrum of PepB, suggesting that disruption of one of the UAU triples destabilized the entire stem 2. The quality of the spectrum of the PepC RNA was too poor to draw any conclusions.

Alphaflexiviridae

Mandarivirus	S1 L1 S2 S1 L2 S2
Indian citrus ringspot (AF406744)	--UUUCAG----UUU-------CCA--------UUUU---CUGAAA-----------------------------AAAAAAAAAA
Citrus yellow vein clearing virus (JX040635)	--UUUCAG----UUU-------CCA--------UUUU---CUGAAA-----------------------------AAAAAAAAAA
**unclassified Mandarivirus**	
Citrus yellow mottle virus (MK957246)	--UUUCAG----UUU-------CCA--------UUUU---CUGAAA-----------------------------AAAAAAAAAA

Potexvirus	S1 ---------------- L1 -------------- S2 S1 ---------------------------- L2 ---------------------------------- S2
Actinidia virus (NC_028649)	----GUGG----UUUU---CUACAGUUUA----UUUU---CCAC----CUACCUAGUAGUU-------------AAAAAAAAAA
Allium virus X (NC_012211)	--AAACAG----UUU----CACUAGAC--------UU---CUGUUU--U---------------------------AAAAAAAAAA
Alstroemeria virus X (NC_007408)	----GGUG----UUUUU--CUACAGUUUUA---UUUU---CACU----AAUAUACUAUGUGUGUGUU--------AAAAAAAAAA
Alternanthera mosaic virus (LC107515)	--UGUGCGG---UUU----AAUACCUA-------UUU--ACGCACA-----------------------------AAAAAAAAAA
Ambrosia asymptomatic virus 1(NC_055542)	--CGCGGUAG--UUU----GUGC-----------UUU--AGCCGCG-----------37^---------------AAAAAAAAAA
Asparagus virus 3 (NC_010416)	---GUGGG----UUU----CUAUAGUUUA-----UUU---CCCAC------------49*---------------AAAAAAAAAA
Babaco mosaic virus (NC_036587)	---UUUGGC---UUU----AAUAUUAG------UUUU---CCAAA------------------------------AAAAAAAAAA
Bamboo mosaic virus (NC_001642)	--UUUUGG----UUU----CUACAG------UUUUUU---CCAAAA-----------------------------AAAAAAAAAA
Cactus virus X (JF937699)	---UUCUG----UUUU---AAUGCAAG-------UUU---CAGAA------------------------------AAAAAAAAAA
Cnidium virus X (NC_055546)	------GG----UUUUU--CCCUG--------UUUUU---CC------UUUUUAAUUAAAAAAA-----------AAAAAAAAAA
Cymbidium mosaic virus (NC_001812)	----GUGG----UUUU---CUAAAGUUUG-----UUU---CCAC----UACUGGCAUAAUAAGUUUAGCCAGAUAAAUAAAAAAA
Euonymus yellow mottle associated virus (NC_055574)	----GCGAG---UUUU---AAUUUUC------UUGUU--AUCGC-------------------------------AAAAAAAAA
Euonymus yellow vein virus (NC_035190)	-CUGACAG----UUU----AAUAGUAG--------UU---CUGUCAGC---------------------------AAAAAAAAAA
Hosta virus X (NC_011544)	---UUUGG----UUU----AACAGAAAA------UUU---CCAAA------------------------------AAAAAAAAAA
Hydrangea ringspot virus (NC_006943)	----UUUG----UUUU---ACAGUUCC-------UUU---CAAA----UUU------------------------AAAAAAAAAA
Lagenaria mild mosaic virus (AB546335)	--UUUUUG----UUUU---AACAGUA-------UUUU---CAAAAA-----------------------------AAAAAAAAAA
Lettuce virus X (NC_010832)	----GUGG----UUUU---CUACAGUUUG----UUUU---CCAC----UUACGCCUUUCUAGCGUAAGU------AAAAAAAAAA
Malva mosaic virus X (NC_008251)	----GUGG----UUU----AUAUAGUUUUA----UUU---CCAC----UUAUGCUAAUAAAGUAUAAUU------AAAAAAAAAA
Narcissus mosaic virus (NC_001441)	----GUGG----UUU----AUAAAGUUUUAC----UU---CCAC----UAUCAAUAAAAUAAACUAUUGAUAUU-AAAAAAAAAA
Nerine virus X (NC_007679)	---AUAAGA---UUU----AACACA--------UUUU--ACUUAU------------------------------AAAAAAAAAA
Opuntia virus X (KY348771)	--UUUCUG----UUUU---AACGCAGC-------UUU---CAGAA------------------------------AAAAAAAAAA
Papaya mosaic virus (NC_001748)	---UUUGG----UUUU---GCAAAUUUCC-----UUU---CCAAA------------------------------AAAAAAAAAA
Pepino mosaic virus SP13(NC_004067)	-----GGG----UUUU---CUACAGUUUA----UUUU---CCU-----AAUUAUUUCUUUGAAAUAA--------AAAAAAAAAA
Pepino mosaic virus CH1 (DQ000984)	-----GGG----UUUU---CUAUAGUUUA----UUUU---CCC-----AAUAAAUUGCUUUUGUAAUUUA-----AAAAAAAAAA
Pepino mosaic virus SAR09 (HQ663890)	-----GGG----UUUU---CUACAGUUUA----UUUU---CCC-----UUAGUAUCUAAAUCUACUAAAU-----AAAAAAAAAA
Pitaya virus X (NC_024458)	---UUCUG----UUUU---AAUGCAAG-------UUU---CAGAA------------------------------AAAAAAAAAA
Potato aucuba virus (NC_003632)	---GGGAG----UUU----CAACAGUUUC-----UUU---CUCCC---UAAUCCCCUGAUUAAAGUGGUUUAAAUAAAAAAAAAA
Potato virus X (NC_011620)	----CCAG----UUU----CAUAGUA-------UUUU---CUGG----UUUGAUUGUAAAAAAUGAAUAAUAUAAAUAAAAAAAA
Scallion virus X (NC_003400)	----GUGG----UUUU---CUACAGUUUA----UUUU---CCAC-------------55**--------------AAAAAAAAAA
Schlumbergera virus X (NC_011659)	---UUCUG----UUUU---AAUGCAAG-------UUU---CAGAA------------------------------AAAAAAAAAA
Senna mosaic virus (NC_030746)	----UUGG----UUUUU--GAACUUA-------UUUU---CCAA-------------95***-------------AAAAAAAAAA
Strawberry mild yellow edge virus (NC_003794)	-----AGG----UUU----GUUUUUC------UUUUU---CCU-----U--------------------------AAAAAAAAAA
Vanilla virus X (NC_035205)	---UUUUG----UUU----CUAAGA--------UUUU---CAAAA------------------------------AAAAAAAAAA
White clover mosaic virus (NC_003820)	--UUUCUG----UUU----AAUAAAA--------UUU---CAGAAA-----------------------------AAAAAAAAAA
Yam virus X (NC_025252)	--AAACCCUC--UUU----GAUAGUA----UUUUUUU---GGGUUU--UU-------------------------AAAAAAAAAA
Zygocactus virus X (NC_006059)	---UUCUG----UUUU---AAUGCACA-------UUU---CAGAA------------------------------AAAAAAAAAA

Unclassified potexvirus	
Paris polyphylla virus X (DQ530433)	---UUUUG----UUU----AAUAAAA--------UUU---CAAAA------------------------------AAAAAAAAAA
Papaya virus X (MN265368)	---UUUUG----UUU----AAUGCCAG-------UUU---CAAAA------------------------------AAAAAAAAAA
Agave potexvirus 1 (MW328740)	---UAGGG----UUU----CAGUUUUAC------UUU---UCCUG------------------------------AAAAAAAAAA

Unclassified allexivirus	
Garlic yellow virus (MN059396)	---UUUGU----UUUU----AAAGGA-----UUUUU---ACAAA-----------------------------AAAAAAAAAAA
Garlic yellow virus (MN059394)	---UUUGU----UUUU----AAAGUA-----UUUUU---ACAAA-----------------------------AAAAAAAAAAA

^UGAACUUCCUUUGCACUGACGUAAUUAGCUAACAACG insert in Ambrosia asymptomatic virus 1

*AGCAGACUAUCAUAUUUACUCUCUUUGAGCGUUAAUAAGUACGUGUGUU insert in Asparagus virus 3

**AUCAGACUCUCCAUCCUACUAGCUUUAUCCGCAUGUAUGAAUGUAAGUUUGUUUU insert in Scallion virus X

***UUCUUGCCACCGCCAGAGUGAGAGUCUAGUUUAGUCAGCCCGUUGUUUUCGCACUUUUGUUGGGGCUAUUGAGUUUUCAAAAGUGCUGUCUAGCU insert in Senna mosaic virus

Betaflexiviridae

Quinvirinae

Banana mild mosaic virus (NC_002729)	-----UGU----UUUU----ACUAAA------UUUU---ACA---------------------------AAAAAAAAAA
Banana virus X (NC_043086)	---UUUGG----UUUU----AAGAAUA-----UUUU---CCAAA-------------------------AAAAAAAAAA
Sugarcane striate mosaic associated virus (NC_003870)	---UUUGG----UUUU----ACAUA-------UUUU--CCCAAA-------------------------AAAAAAAAAA

Carlaviruses	
Aconitum latent virus (NC_002795)	---UGAAG----UUUU----AUCUA------UUUUU--CCUUUA-------------------------AAAAAAAAAA
American hop latent virus (JQ728538)	---UUAAG----UUUU----AAAUA------UUUUU--CCUUAA-------------------------AAAAAAAAAA
Atractylodes mottle virus (KR34934)	---UGAGG----UUUU----AAGAUA-----UUUUU--CCCUUA-------------------------AAAAAAAAAA
Blueberry scorch virus (AY941199)	----UUUG----UUUUU---AAACUA-----UUUUU--GCAAA--------------------------AAAAAAAAAA
Butterbur mosaic virus (NC_013527)	---CUCUC----UUUU----AUAUA-------UUUU--GGAGAGGUUAAU-------------------AAAAAAAAAA
Carnation latent virus (AJ010697)	-AAAUGCG----UUUU----AAAUA------UUUUU--CCGUAUUUU----------------------AAAAAAAAAA
Chrysanthemum virus B (AB245142)	---GUGAG----UUUU----AAAAUA-----UUUUU--CCUUACUU-----------------------AAAAAAAAAA
Chrysanthemum virus R (NC_040703)	----GAAG----UUUU----AAAGUA-----UUUUU--CCUUCUUU-----------------------AAAAAAAAAA
Cole latent virus (AY340584)	---UAUGG----UUUUU---AAGCUA-----UUUUU--AUCCUAAG-----------------------AAAAAAAAAA
Coleus vein necrosis virus (NC_009764)	--UUUCUG----UUUU----AAUAUA-----UUUUU--AUGGAAA------------------------AAAAAAAAAA
Cowpea mild mottle virus (NC_014730)	---ACCGG----UUUU----AAAGUUA-----UUUU--CCUGGUUUU----------------------AAAAAAAAAA
Cucumber vein-clearing virus (JN591720)	-AAUACUG----UUUUU---AAUAAUA----UUUUU---CAGGUU------------------------AAAAAAAAAA
Daphne virus S (NC_008020)	----UAAG----UUUU----AAAUA------UUUUU--CCUUA--------------------------AAAAAAAAAA
Elderberry carlavirus C (NC_029087)	----AAAG----UUUU----AAUGUA-----UUUUU-CCCUUUCUU-----------------------AAAAAAAAAA
Elderberry carlavirus D (NC_029088)	---UUUGC----UUUU----AACUA------UUUUU---GCAAA-------------------------AAAAAAAAAA
Elderberry carlavirus E (NC_029089)	----UAAG----UUUU----AAUAUA-----UUUUU-ACCUUA--------------------------AAAAAAAAAA
Gaillardia latent virus (KJ415259)	----UAAG----UUUU----AAUAUA-----UUUUU--CCUUA--------------------------AAAAAAAAAA
Garlic common latent virus (NC_016440)	----AAGG----UUU-----AAUAUA-------UUU---CCUUUU------------------------AAAAAAAAAA
Helenium virus S (D10454)	----UAAG----UUUU----AAUAUA-----UUUUU--CCUUA--------------------------AAAAAAAAAA
Helleborus mosaic virus (FJ196838)	---UUUGC----UUUU----AAAUA-------UUUU--GGCAAA-------------------------AAAAAAAAAA
Helleborus net necrosis virus (NC_012038)	---UUUGC----UUUU----AAUAUA-----UUUUU---GCAAA-------------------------AAAAAAAAAA
Hippeastrum latent virus (NC_011540)	----UAGU----UUUU----AAAUA-----UUUUUU---ACUA--------------------------AAAAAAAAAA
Hop latent virus (NC_002552)	--UUUGGU----UUUU----AAAUA-------UUUU--CACCAAA------------------------AAAAAAAAAA
Hop mosaic virus (NC_010538)	--UUUGGU----UUUU----AAAUA------UUUUU--AGCCAAA------------------------AAAAAAAAAA
Hydrangea chlorotic mottle virus (NC_012869)	--UUUCUG----UUUU----AAAACUA----UUUUU--CCAAAAA------------------------AAAAAAAAAA
Kalanchoe latent virus (NC_013006)	--UUUGUU----UUU-----AAAUA-------UUUU---AGCAAA------------------------AAAAAAAAAA
Ligustrum necrotic ringspot virus (NC_010305)	---UUUGU----UCUU----AAACUA------UUUU--CGCAAA-------------------------AAAAAAAAAA
Ligustrum virus A (NC_031089)	--UUUAAG----UUUUU---AAAUA------UUUUU--ACUUAAA------------------------AAAAAAAAAA
Lily symptomless virus (NC_005138)	---UUUGU----UUUU----AAAA--------UUUU--AGCAGA-------------------------AAAAAAAAAA
Lily latent virus (AJ131812)	---UUUGU----UUUU----AAAUA-------UUUU--AGCAAA-------------------------AAAAAAAAAA
Melon yellowing-associated virus (LC224308)	--UUUCGG----UUUU----AAGAUA-----UUUUU--CCCGAAA------------------------AAAAAAAAAA

Mirabilis jalapa mottle virus (NC_016080)	---UUUGU----UUUU----AACUA------UUUU--AGCAAA--------------------------AAAAAAAAAA
Narcissus common latent virus (NC_008266)	---UUUGG----UUUUU---AAGCUA---UUUUU--CCCAAA--------------------------AAAAAAAAAA
Narcissus symptomless virus (NC_008552)	----UUGG----UUUU----AUCGAUAA-----UUU--ACCAAU--------------------------AAAAAAAAAA
Nerine latent virus (NC_028111)	----UUGG----UUUU----AUCGAUAA-----UUU--ACCAAU--------------------------AAAAAAAAAA
Passiflora latent virus (NC_008292)	---UUUGU----UUUU----AAAAGA-----UUUU--AGCAAA--------------------------AAAAAAAAAA
Pea streak virus (KP828803)	-CAAGUGUG---UUUU----AAUAUA-----UUUUU--AAUGCUUGU-----------------------AAAAAAAAAA
Phlox virus B (NC_009991)	--GAGAAG----UUUU----AAUAUA-----UUUU---CUUUUU--------------------------AAAAAAAAAA
Phlox virus M (FJ159381)	-----UGG----UUUUU---AAGCUA-----UUUUUAUCCUAAA--------------------------AAAAAAAAAA
Phlox virus S (EF492068)	----AAAG----UUUU----AAGAUA-----UUUUU--ACUUUUAU------------------------AAAAAAAAAA
Poplar mosaic virus (NC_005343)	---UUUGGA---UUUUU---AAUAUA------UUUU-AACCAAA--------------------------AAAAAAAAAA
Potato latent virus (NC_011525)	---UAAAG----UUUU----AAAUA------UUUUU--CCUUUA--------------------------AAAAAAAAAA
Potato virus H(NC_018175)	----UUAG----UUUU----AAAUA------UUUUU--CCUAA---------------------------AAAAAAAAAA
Potato virus M (NC_001361)	---UUGGU----UUUU----AACUA------UUUUU--AGCCAA--------------------------AAAAAAAAAA
Potato virus P (EU338239)	---UUUGG----UUUUU---AACCUA------UUUU--ACCAAA--------------------------AAAAAAAAAA
Potato rough dwarf virus (NC_009759)	----UUUG----UUUUU---AAACUA------UUUUAGCCAAA---------------------------AAAAAAAAAA
Potato virus S (JQ647830)	----AUGU----UUUU----AAAAUA------UUUU--AGCAUU--------------------------AAAAAAAAAA
Red clover vein mosaic virus (KR108251)	-----AAG----UUUU----AAUAUA-----UUUUU--CCUUC---------------------------AAAAAAAAAA
Shallot latent virus (LC279526)	---UUUGU----UUUU----AAAAUA------UUUU--CGCAAA--------------------------AAAAAAAAAA
Sweet potato C6 virus (JQ814719)	--AAUGGU----UUUU---CAUUAUUAA----UUUU--AGCCUAUUAAGAGCCAACGAGCUCCUCUGGAAAAAAAAAAAA
Sweet potato chlorotic fleck virus (NC_006550)	---UUUGCAUG-UUUU----AAAGUAC-----UUUU-GAGCAAA--------------------------AAAAAAAAAA
Sweet potato yellow mottle virus (KR072674)	---UUUGCAUG-UUUU----AAAGUAC-----UUUU-GAGCAAA--------------------------AAAAAAAAAA
Yam latent virus (NC_026248)	---UUGGU----UUUU----AACUA------UUUUU--GGCCAA--------------------------AAAAAAAAAA
**unclassified Carlavirus**	
Allium carlavirus A (MH898470)	--UUUGUU----UUUU----AAAUA-------UUUU---AACAAA-------------------------AAAAAAAAAA
Birch carlavirus (MH536506)	----AAAG----UUUU----AAAUA------UUUUU-ACCUUUU--------------------------AAAAAAAAAA
Cactus carlavirus 1 (MK415316)	---UUUGU----UUUUU---AAAAUA------UUUU--AGCAAA--------------------------AAAAAAAAAA
Cactus carlavirus 2 (MK415317)	---UUUGU----UUUU----AAUAUA-----UUUUU---GCAAA--------------------------AAAAAAAAAA
Carrot carlavirus WM-2008 (EU881919)	--GUAAGU----UUU-----AUCUA-------UUUU---GCUUAU-------------------------AAAAAAAAAA
Cole mild mosaic virus (MK684348)	-----GGU----UUUU----AAGCUA-----UUUUU---AUCCU--------------------------AAAAAAAAAA
Elderberry carlavirus A (NC_029085)	---GUAAG----UUUU----AAUCUA-----UUUUU--CCUUAU--------------------------AAAAAAAAAA
Elderberry carlavirus B (NC_029086)	----UAAG----UUUU----AUCUA-------UUUU-ACCUUA---------------------------AAAAAAAAAA
Jasmine virus C (NC_030926)	--UUUGUG----UUUU----AACUA------UUUUU--CCGCAAA-------------------------AAAAAAAAAA
Magnolia carlavirus (JX912499)	---UUUGU----UUUU----AAACUA------UUUU--AGCAAA--------------------------AAAAAAAAAA
Opuntia virus H (KU854930)	---UUUGU----UUUU----AAAAUA------UUUU--AGCAAA--------------------------AAAAAAAAAA
Pepper virus A (NC_034376)	-----CGG----UUU-----AACUA--------UUU--CCUG----------------------------AAAAAAAAAA
Red clover carlavirus 1 (MG596239)	---AAAGU----UUUU----AAUAUA------UUUU--CGCUUUU-------------------------AAAAAAAAAA
Red clover carlavirus A (KY474546)	----UAAG----UUUU----AAAUA------UUUUU--CCUUA---------------------------AAAAAAAAAA
Rose virus A (MN053272)	-AAGAAAG----UUUU----AAUAUA-----UUUUU--CCUUUUUU------------------------AAAAAAAAAA
Rose virus B (MT473961)	----UAAG----UUUU----AAUAUA-----UUUUU--CCUAA---------------------------AAAAAAAAAA
Sedum latent virus (FJ560901)	---UUUGU----UUUU----AAAAGA------UUUU--AGCAAAAAAAAAAAACUUGGUUCUCAA-----AAAAAAAAAA
Soybean carlavirus 1 (MW176107)	----AAGU----UUUU----AACUA------UUUUU---ACUU---------------------------AAAAAAAAAA
Stevia carlavirus 1 (MW328723)	----UAAG----UUUU----AAUAGA-----UUUUU--CCUUA---------------------------AAAAAAAAAA
Tagetes carlavirus 1 (MW328722)	----AAAG----UUUU----AAUAUA-----UUUUU---CUUA---------------------------AAAAAAAAAA
Whitefly transmitted carlavirus (MK910291)	-GAACCGG----UUUU----AAAGUUA-----UUUU--CCUGGUUUC-----------------------AAAAAAAAAA
**Foveavirus**	
Apple stem pitting virus (NC_003462)	--UUUUUG----UUUU----AACUAGA-----UUUU---CAAAAA-------------------------AAAAAAAAAA
Apricot latent virus (NC_014821)	--UUUUUG----UUUU----AACUAGA-----UUUU---CAAAAA-------------------------AAAAAAAAAA
Pear vein yellows virus (D21828)	--UUUUUG----UUUU----AACUAGA-----UUUU---CAAAAA-------------------------AAAAAAAAAA
Peach sooty ringspot virus (AF318062)	--UUUUUG----UUUU----AACUAGA----UUUUU---CAAAAA-------------------------AAAAAAAAAA
Peach asteroid spot virus (AF318061)	--UUUUUG----UUUU----AACUAGA------UUU--CCAAAAA-------------------------AAAAAAAAAA
Grapevine rupestris stem pitting associated (MG938307)	UUUGUGUG----UUUUU---AUAGA-------UUUU---CACUCAAA-----------------------AAAAAAAAAA
Grapevine virus T (MH674185)	----UGUG----UUUU----AAUAUA-----UUUUU--CCGCA---------------------------AAAAAAAAAA
Grapevine virus T (MH802011)	----UGCG----UUUU----AAUAUA-----UUUUU--CCGUA---------------------------AAAAAAAAAA
Peach chlorotic mottle virus (NC_009892)	--UUUUGGC---UUU-----AAUAUA------UUUU---CCAAAA-------------------------AAAAAAAAAA
Rubus canadensis virus 1 (NC_019025)	-UUUUUGG----UUCUUUU---AAC-----UUUUUU---CUAAAAA------------------------AAAAAAAAAA
Asian prunus virus 2 (NC_028868)	---UUUGC----UUUUU---AUCGCUAA---UUUUU---GCAAA--------------------------AAAAAAAAAA
Asian prunus virus 1 (NC_025388)	---UUUGC----UUUUU---AUCGCUAA----UUUU---GCAAA--------------------------AAAAAAAAAA
**unclassified Foveavirus**	
Apple green crinkle associated virus (HE963831)	--UUUUUG----UUUU----AACUAGA-----UUUU---CAAAAA-------------------------AAAAAAAAAA
Asian prunus virus 3 (DQ205238)	---UUUGC----UUUUUU--AUCGCUAA---UUUUU---GCAAA--------------------------AAAAAAAAAA
Camellia ringspot associated virus 4 (MT028514)	---UUUUG----UUUU----AAAAUA-----UUUUU--CCAAAA--------------------------AAAAAAAAAA
Cherry virus B (LC373513)	-UUUGUGU----UUUU---AAUCGAUAA---UUUUU--AACUAAA-------------------------AAAAAAAAAA
Garlic yellow stripe associated virus (MT981417)	--UUUGUG----UUUUU----AUAUA-----UUUUU--CCGCUAAA------------------------AAAAAAAAAA
Rubus virus 1 (MN944023)	----UAAG----UUUU-----AAAUA-----UUUUU-GUCUUA---------------------------AAAAAAAAAA
**Robigovirus**	
African oil palm ringspot virus (NC_012519)	----UUGCAUG-UUUU----AAUAUA--------UUU-GAGCAA--------------------------AAAAAAAAAA
Cherry green ring mottle virus (NC_001946)	----AGGG----UUUU----AAAAUA------UUUU---CCUUCAGUUUCCUUAGAGAAA----------AAAAAAAAAA
Cherry necrotic rusty mottle virus (NC_002468)	----AGGG----UUUU----AAUAUA------UUUU---CCUUCAGUUUUCUACGAGAGAA---------AAAAAAAAAA
Cherry twisted leaf associated virus (NC_024449)	----AGGG----UUUU----AAUAUA------UUUU---CCUUUAGUUUUCUACAUCUUUUUU-------AAAAAAAAAA
Cherry rusty mottle associated virus (NC_020996)	----AGGG----UUUU----AAUAUA------UUUU---CCUUUAGUUUGCUAUGCAAA-----------AAAAAAAAAA
**unclassified Robigovirus**	
Cherry virus Turkey (MK600387)	----AAGG----UUU-----AAUAGA--------UUU---CCUUUGGCUUUAAAAAGUACUUUUUCUUUUAAAAAAAAAAA
Cherry Robigo virus 5 (MK035727)	-----AGG----UUU-----AAUAGA--------UUU---CCUAUGGCUUUAGAAGUAAUUUUUCUUUUUAAAAAAAAAAA
Pomes virus (MW331541)	----AAGG----UUU-----AAUAUA--------UUU---CCUUUG------------------------AAAAAAAAAAA
**Unclassified Quinvirinae**	
Dioscorea virus A (LC467961)	---UUUUG----UUUUU---AAAAGAA-----UUUU---CAAAA--------------------------AAAAAAAAAA
Papaya mild mottle associated virus (MK984598)	---UUUGG----UUUU----AAAGUA------UUUU---CCAAA--------------------------AAAAAAAAAA

Trivirinae

**Capillovirus**	
Apple stem grooving virus (NC_001749)	---CCCGG----UUUU-----CUGAGCA-----UUU---CCGGG------------39****--------AAAAAAAAAA
Citrus tatter leaf virus (JQ765412)	---CCCGG----UUUU-----CUGUGCA-----UUU---CCGGG------------40*****-------AAAAAAAAAA
Pear black necrotic leaf spot virus (AY596172)	---ACCCGG---UUUU-----CUGAGCA-----UUU---CCGGGU-----------39******------AAAAAAAAAA
Cherry virus A (NC_003689)	----AGGAC---UUUUU----AUUC-------UUUU---UCCUU--------------------------AAAAAAAAAA
Mume virus A (NC_040568)	----AGGAC---UUU------AUUUCC-----UUUU---UCCUU--------------------------AAAAAAAAAA
Currant virus A (NC_029301)	---AAGGAG---UUU------AUGAUC------UUU---UCCUUUUUUGAAAAAAAA-------------AAAAAAAAAA
**Unclassified Capillovirus**	
Avellana capillovirus 1 (MW328729)	---UACUAG---UUUU-----CAAAAA------UUU---CUAGUAGCAAGAGCCAAGGCUCC--------AAAAAAAAAA
Breadfruit capillovirus 1 (MW328738)	---CCCGG----UUUU-----CUGUGAC-----UUU---CCGGG------------39*******-----AAAAAAAAAA
Rubber tree capillovirus (MN047299)	---UUUGCAG--UUUUU----AGUA-------UUUU-AAGCAAA--------------------------AAAAAAAAAA
Yacon virus A (NC_030657)	---UAUAG----UUUU-----GCGUAUA-----UUU---CUAUA--------------------------AAAAAAAAAA
**Unclassified Chordovirus^^**	
Hogweed virus 4 (OK032418)	---UCUAG----UUUU-----AUAUAC-----UUUU---CUAGAAAAU----------------------AAAAAAAAAA
**Citrivirus**	
Citrus leaf blotch virus (NC_003877)	---UGUCU----UUU------AAGAAC-----UUUU---AGACA--------------------------AAAAAAAAAA
Dweet mottle virus isolate (FJ009367)	---UGUCU----UUU------AAGAAC-----UUUU---AGACA--------------------------AAAAAAAAAA

**Unclassified Citrivirus**	
Avocado citrivirus 1 (MW328736)	-----GGU----UUU------AACGGAAA----UUU---ACU-----------------------------AAAAAAAAAA
Citrivirus sp. strain BJ (MN253488)	---UGUCU----UUU------AAGAAC-----UUUU---AGACA---------------------------AAAAAAAAAA
Paeonia citrivirus (MH898501)	---UGUCU----UUU------AAGAAC-----UUUU---AGACA---------------------------AAAAAAAAAA
Haruka-associated citrivirus (MH144344)	---UGUCU----UUU------AAGAAC-----UUUU---AGACA---------------------------AAAAAAAAAA
Haruka-associated citrivirus CN-2 (MH558590)	---UGUCG----UUU------AAGAAC-----UUUU---GGACA---------------------------AAAAAAAAAA
Nandina citrivirus (MN055483)	---UGUCU----UUU------AAGAAC-----UUUU---AGACA---------------------------AAAAAAAAAA
**Divavirus**	
Diuris virus A (NC_019029)	----GCAG----UUUUU----CUUUGCGC---UUUU---CUGUUGUUUUCUUAAGUAUUUUCUUAAAAAAAAAAAAAA
Diuris virus B (NC_019030)	--CAUGGG----UUUUU----AUGCAUA-----UUU---CCUUUG------------52********-----AAAAAAAAA
Hardenbergia virus A (NC_015395)	--CAUGGG----UUUUU----CUUGUUA----UUUU---CCUUUGUUUUCUUCUUUUGCUUUUUUCCGAAGAAAAAAAAA
**Unclassified Divavirus**	
Ocimum basilicum RNA virus 1 (NC_035462)	--CAUGGG----UUUUU----CUGAAA-----UUUU---CCUUUGUUUUUCUUAAAAAA-----------AAAAAAAAAA
**Prunevirus**	
Actinidia seed-borne latent virus (NC_040800)	---UUUGGACG-UUU------AAAAA-------UUU-GACCAAA---------------------------AAAAAAAAAA
Apricot vein clearing associated virus (MK170159)	---UGUCU----UUUU-----AAAGAAA-----UUU---AGACA---------------------------AAAAAAAAAA
Caucasus prunus virus (NC_038325)	---UGUCU----UUUU-----AAAUA------UUUU---AGACA---------------------------AAAAAAAAAA
**Ravavirus**	
Ribes americanum virus A (NC_040797)	---AUGAG----UUUU-----AAAA--------UUU---CUUAUUUGAAAAAUUAUUCU-----------AAAAAAAAAA
**Tepovirus**	
Potato virus T (NC_011062)	---UUUGCAUGGUUUUU----CAAG------UUUUUAAAGCAAA---------------------------AAAAAAAAAA
Prunus virus T (NC_024686)	--UUUCUG----UUU------AAGAA------UUUU---CAGAAA--------------------------AAAAAAAAAA
**Unclassified tepovirus**	
Agave virus T (MW323519)	--UUUUCCAGUGUUUU-----CGAC-------UUUUGAAGGAAAA--------------------------AAAAAAAAAA
Cherry virus T (MT090966)	-UUUUUCG----UUUU-----ACUGAUAA---UUUU---CGAAAAA-------------------------AAAAAAAAAA
Ficus tepovirus A (MH898491)	-UUUUUCG----UUUU-----ACUGAUAA---UUUU---CGAAAAA-------------------------AAAAAAAAAA
Tricosanthes tepovirus A (MH898525)	--AAAGUUAC--UUUU-----AAGAA----UUUUUU--AAACUUUU------------------------AAAAAAAAAA
Zostera virus T (MK514426)	----UUCU----UUU------AACGAAUAA---UUU---AGAA---------------------------AAAAAAAAAA
**Trichovirus**	
Apple chlorotic leaf spot virus (NC_001409)	---UGUAGAC--UUUU-----AAAUA------UUUU--ACUACA---------------------------AAAAAAAAAA
Apricot pseudo-chlorotic leaf spot virus (NC_006946)	-----UAGAC--UUUU-----AAAUA------UUUU---CUAC-----------------------------AAAAAAAAAA
Cherry mottle leaf virus (NC_002500)	---UAUAGAC--UUUU-----AAAUA------UUUU---CUAUA---------------------------AAAAAAAAAA
Grapevine berry inner necrosis virus (NC_015220)	----UUUG----UUUU-----AAUUAA-----UUUU---CAAA----------------------------AAAAAAAAAA
Grapevine Pinot gris virus (NC_015782)	----UUUG----UUUU-----AAUGAA-----UUUU---CAAA----------------------------AAAAAAAAAA
Peach mosaic virus (DQ117579)	---UGUAGAC--UUUU-----AAAAA-------UUU--ACUGCA---------------------------AAAAAAAAAA
Phlomis mottle virus (AM920542)	----UGUG----UUUU-----AAUAUA------UUU--ACACAG----------------------------AAAAAAAAAA
**unclassified Trichovirus**	
Cherry symptomless virus (MK770441)	---UAUAGAC--UUUU-----AAAUA------UUUU--ACUAUAU--------------------------AAAAAAAAAA
Mexico trichovirus (MK012336)	---UUCAGAC--UUUU-----AAAUA------UUUU--ACUGAA---------------------------AAAAAAAAAA
Peach chlorotic leaf spot virus (MH084696)	---UAUAGAC--UUUU-----AAAUA------UUUU--ACUAUAU--------------------------AAAAAAAAAA
**Wamavirus**	
Watermelon virus A(MK292710)	---ACGGGG---UUUU-----ACA-------UUUUU--ACUCGUUUGCUUAGCAA---------------AAAAAAAAAA
**unclassified Betaflexiviridae**	
Agapanthus virus A (MT533609)	-UUAAAAG----UUU------AAGAAA-----UUU---CUUUUAA-------------------------AAAAAAAAAA
Camellia japonica ass. Betaflexivirus 1 (MN532565)	--AUGCCU----UUU------CAAUA-------UUU--AGGGCAU--------------------------AAAAAAAAAA
Camellia japonica ass. Betaflexivirus 2 (MN385582)	----UGUC----UUUU-----AUGAAUA-----UUU-AUGACA----------------------------AAAAAAAAAA
Camellia japonica ass. Betaflexivirus 3 (MN532566)	----UGUC----UUUU-----AUGAAUA-----UUU-AUGACA----------------------------AAAAAAAAAA
Camellia ringspot associated virus 2 (MK050793)	----UGUC----UUUU-----AUGAAUA-----UUU-AUGACA----------------------------AAAAAAAAAA
Camellia ringspot associated virus 3 (MK050796)	---UUUCU----UUU------AAUGUUAA----UUU---AGAAA---------------------------AAAAAAAAAA
Citrus chlorotic leaf spot virus (MN879752)	---GAGAG----UUUU-----AAUAA------UUUU---CUUUU---------------------------AAAAAAAAAA
Gymnadenia betaflexivirus 1 (MW328732)	---AAGGG----UUUUU----AUGUUCA-----UUU---CCUUUGUUUGUUCUUUUUUUUUUUUAGGACAAAAAAAAAA
Lettuce chordovirus 1 (NC_040627)	--UGAGGU----UUUU-----AUCGAUAAA---UUU---ACCUUAUU------------------------AAAAAAAAAA
Loquat virus A (MK936045)	-UUUAAAG----UUUU-------UCA------UUUU---CUUUAAA-------------------------AAAAAAAAAA
Panax ginseng flexivirus 1 (NC_040643)	--UUUCGU----UUU------ACUAUA----UUUUU---GCGAAA--------------------------AAAAAAAAAA
Peony betaflexivirus 1 (MN253489)	---AGUUG----UUUU-----AAUUUA-----UUUU---CAAUUUU-------------------------AAAAAAAAAA
Shallot virus S (MH292861)	---UUUGU----UUUU-----AAAAUA-----UUUU--CGCAAA---------------------------AAAAAAAAAA
Sichuan betaflexivirus 1 (MW897301)	----UUUG----UUUUU----AAAUA------UUUU---CGAA----------------------------AAAAAAAAAA
Teosinte-associated betaflexivirus (OK018179)	--UUUAAG----UUUU-----AAAAA------UUUU---CUUAAA--------------------------AAAAAAAAAA
**Unclassified virus**	
Papaya mottle associated virus (MK984601)	-AAUCUGU----UUUU-----AAAGUA----UUUUU---AUGGAUUU------------------------AAAAAAAAAA

****UUAGUGUGGUUUUUCUAGAGUCUAGAGUUUGUCCACUCU insert Apple stem grooving virus
*****UUAGUGUGGUUUUUUCUAGAGUCUAGAGUUUGUCCACUCG insert Citrus tatter leaf virus
******UUAGUGUGGUUUUUUCUAGAGUCUAGAGUUUGUCCAUUCU insert Pear black necrotic leaf spot virus
*******UUUGAACGUUUGCUCUAGAGUCUAGGGAUUGUCCGUCUC insert Breadfruit capillovirus 1
********UUUGUUUGAUGUUUGUUUGUUUGGUCAAAAAAAGGAAAUAUGCAUAAAAACC insert Diuris virus B
^^The only two members of the chordovirus genus, Carrot betaflexivirus 1 and 2, have incomplete 3′ends.

Fig. S2. Alignment of the 3′-ends of Alpha- and Betaflexiviridae RNAs

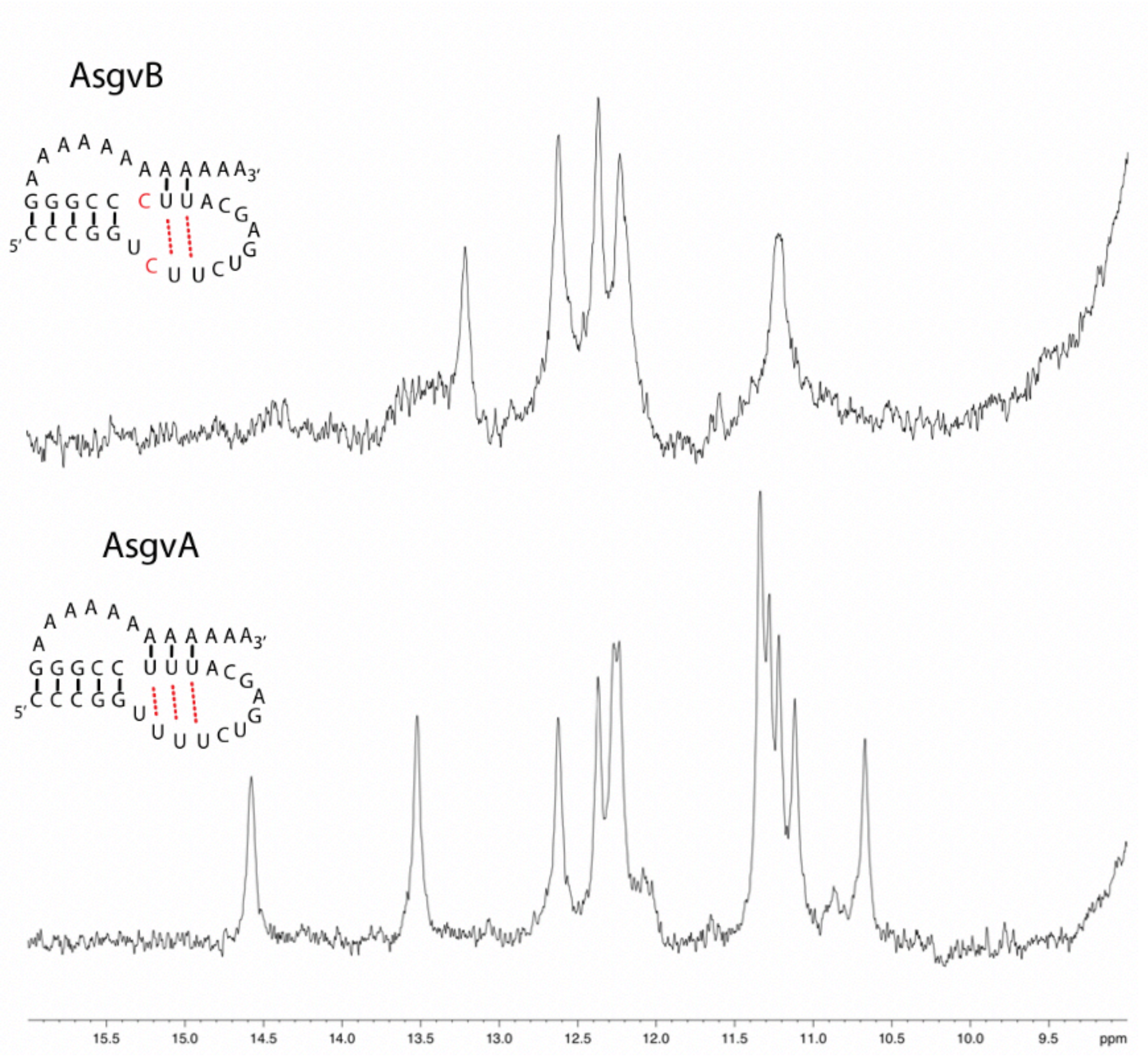

Fig. S3. 1D proton NMR of AsgvA and AsgvB. Peaks downfield of 13.5 ppm in the spectrum of AsgvA are tentatively assigned to AU base pairs of stem 2. These resonances are absent in the spectrum of AsgvB, suggesting that it does not form stem 2.

**Group 1.**	
Plantago asiatica mosaic virus (NC_003849)	GCCU-ACGACCAGUGAAAAUCUGGU-GGGCCCAAA
Plantago asiatica mosaic virus (KU159090)	GCUU-ACGACCAGUGAAAGUCUGGU-GAGCCCAAA
Tulip virus X (NC_004322)	GCCCUACAACCAGUGAAAGUCUGGUGGGGCCAAAA
Tulip virus X (AY842510)	GCCCUUCAACCAGUGAAAGUCUGGUGGGGCCAAAA
**Marafivirus**	
Medicago sativa marafivirus 1 (MF443260)	GGUU-UCAACCAGUGAAAAUCUGGU-GGCCCCUUCA
Alfalfa virus F (NC_040565)	GUCU-UCAACCAGUGAAAAUCUGGU-GGACCCUUCA
**Group 2.**	
Lily virus X (NC_007192)	---AACUCCCAAGAGAGUUAAAAA
Mint virus X (NC_006948)	----UACCCCAAGAGGUAAAAAAA
Phaius virus X (NC_010295)	--CAUGCUCGACGAAGCAUGAAAA
**Allexivirus**	
Arachis pintoi virus (NC_032104)	---GGGUCCCGAGAGAUCCCCCAA
Garlic virus A (MN059325)	---UUGUCCCCAGAGACAAAAAAA
Garlic virus A (MN059300)	---UUGUCCCCAGUGACAAAAAAA
Garlic virus D	--UUUGUCCCAAGUGACAAAAAAA
Garlic virus E (NC_004012)	--UUUGUCCCAUGUGACAAAAAAA
Garlic virus E (MN059326)	--UUUGUCCCAUGCGACAAAAAAA
Alfalfa virus S (MT094142)	-UUUGGUUCUACGCAACCAAAAAA
**Botrexvirus**	
Botrytis virus X (NC_005132)	UUUUUGUCCCAUGCGACAAAAAAA
**Bromovirus**	
Brome mosaic virus (NC_002028)	----AGGACAUAGAUCUU------
**Group 3-5.**	
Clover yellow mosaic virus (NC_001753)	-----ACCCG-UAGACUUU-UGGGUGUAUA
Tamus red mosaic virus (NC_016003)	----UUUGCC-CGAACUUUAGGCAAAAAAA
Cassava mosaic virus X (U23414)	------CCGCGGUCAGUUUGGCGGUUUAAA
Cassava mosaic virus X (MT279196)	------CCGCGAACAGUUUAGCGGUUUAAA
Cassava virus X (KY288487)	---UGUUGCC--CAGGUAUAGGCAACAAAA
Cassava Colombian symptomless virus	---AUAGGUC--CAGGAGGAGACCUAUAAA
Foxtail mosaic virus (NC_001483)	----GGGUGA-AUGCACACAUCGCUUAUAA
Turtle grass virus X (NC_040644)	---GGGGUGA-AUGCACACAUCACUUUAAA
**Vitivirus** classified & unclassified	
Grapevine virus A (JX559641)	--UUGUCAC---ACG---GUGACAAAA
Heracleum latent virus (X79270)	------AAC---ACG---GUUAAAAAA
Grapevine virus A (MZ440721)	--UUGUCAC---AAG---GUGACAAAA
Mint virus 2 (AY913795)	-----AUCC---AAG---GGAUAAAAA
Grapevine virus A (MT070963)	--UUGUCAU---ACU---AUGACAAAA
White ash mosaic virus (NC_011533) unclass.	-CCCAGUCC---ACU---GGACUGGGA
Blackberry virus E (NC_015706) unclass.	----GGGUC-CCACGU--GACCCAAAA
Cherry green ring mottle virus (NC_001946) uncl.	-----UUUC-CUUAGA--GAAAAAAAA
Grapevine virus E (GU903012)	-GAAACGGU---ACAU--ACCGUUUUA
Cherry necrotic rusty mottle virus (NC_002468) uncl.	---UUUUCU--ACGAG--AGAAAAAAA
Grapevine virus B (NC_003602)	-----CGUG-AAUAAA--CACGGAAAA
Grapevine virus D (JQ031715)	----UUUCCAUAAGUAGAGGAAAAAAA
Grapevine virus F (NC_018458) uncl.	---UUGUCC-ACAUGGG-GGACAAAAA
Actinidia virus A (JN427014) uncl.	------GUG--AAUAAA-CACAAAAAA
Actinidia virus B (NC_016404) uncl.	------GUG--AAUAAA-CACAAAAAA
Agave tequilana leaf virus (DQ525858) uncl.	-----CGUC--AAUAG--GGCGAAAAA
Fig latent virus 1 (FN377573) uncl.	------UGG--AGGCAA-CCAAAAAAA

Fig. S4. Alignment of the 3’-ends of Alpha- and Betaflexiviridae that do not adopt a pseudoknot.

Supplementary Table S1: PCR PRIMERS

T7pep	CGAAATTAATACGACTCACTATAGAAAACAAAATAAATA
SP6FLU	CCATTAAGCTTATTTAGGTGACACTATAGAAGAGATCACTAGAAGCTAGCAAAAGCAGGG
pep1	TTTTTTTTTTTTTTTTTTTTTTTTTATTTCAAAGAAATAATTGGG
PepA2	TTATTTCAAAGAAATAATTGGG
PepA6	TTTTTTATTTCAAAGAAATAATTGGG
PepA9	TTTTTTTTTTATTTCAAAGAAATAATTGGG
PepA12	TTTTTTTTTTTTTATTTCAAAGAAATAATTGGG
PepA15	TTTTTTTTTTTTTTTTATTTCAAAGAAATAATTGGG
PepA18	TTTTTTTTTTTTTTTTTTTATTTCAAAGAAATAATTGGG
Pep2	TTTTTTTTTTTTTTTTTTTTTTTTTATTTCAAAGAAATAATTGGGAAAATAAACTGTAGAAAACGCCACTCTGATTAAG
Pep3	TTTTTTTTTTTTTTTTTTTTTTTTTATTTCAAAGAAATAATTGCGAAAATAAACTGTAGAAAACGCCACTCTGATTAAG
Pep4	TTTTTTTTTTTTTTTTTTTTTTTTATTTCAAAGAAATAATTCCAGAAAATAAACTGTAGAAAACTGGCACTCTGATTAAG
Pep5	TTTTTTTTTTTTTTTTTTTTTTTTTATTTCAAAGAAATAATTGGGGAAATAAACTGTAGAAGACCCCACTCTG
Pep6	TTTTTTTTTTTTTTTTTTTCTTTTTATTTCAAAGAAATAATTGGGGAAATAAACTGTAGAAGACCCCACTCTG
Pep7	TTTTTTTTTTTTTTTTTTTTTTTTTATTTCAAAGAAATAATTGGGAGAATAAACTGTAGAGAACCCCACTCTG
Pep8	TTTTTTTTTTTTTTTTTTCTTTTTTATTTCAAAGAAATAATTGGGAGAATAAACTGTAGAGAACCCCACTCTG
Pep9	TTTTTTTTTTTTTTTTTTTTTTTTTATTTCAAAGAAATAATTGGGAAGATAAACTGTAGGAAACCCCACTCTG
Pep10	TTTTTTTTTTTTTTTTTCTTTTTTTATTTCAAAGAAATAATTGGGAAGATAAACTGTAGGAAACCCCACTCTG
Pep11	TTTTTTTTTTTTTTTTTTTTTTTTTATTTCAAAGAAATAATTGGGGGAATAAACTGTAGAGGACCCCACTCTG
Pep12	TTTTTTTTTTTTTTTTTTCCTTTTTATTTCAAAGAAATAATTGGGGGAATAAACTGTAGAGGACCCCACTCTG

pep1 pA2 pA6 pA9 pA12 pA15 pA18

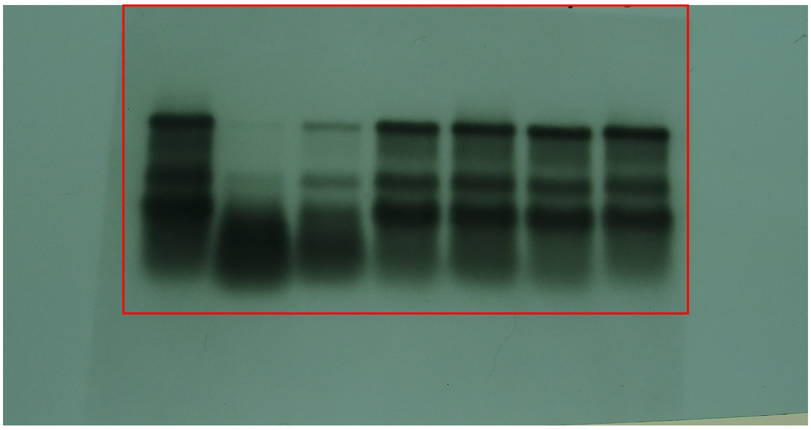

Northern blot showing the accumulation of PepMV gRNA and sgRNAs in protoplasts. Pep1: wt PepMV with A25, A2, A6, A9, A12, A15, A18.

pep1 pep2 pep3

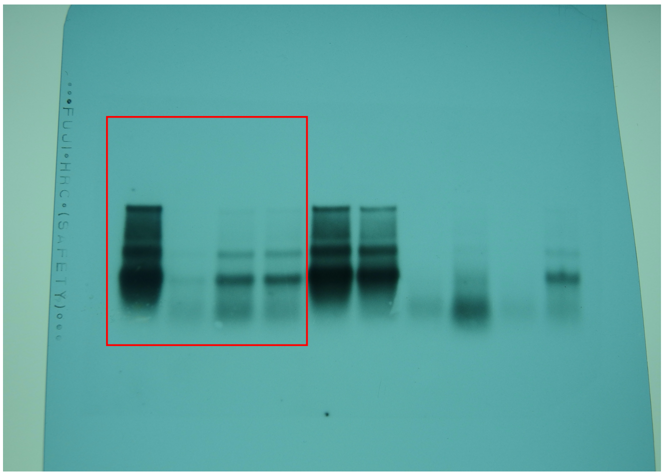

Northern blot showing the accumulation of PepMV gRNA and sgRNAs in *N. benthamiana* protoplasts.

pep1 pep5 pep6 pep7 pep8 pep11 pep12

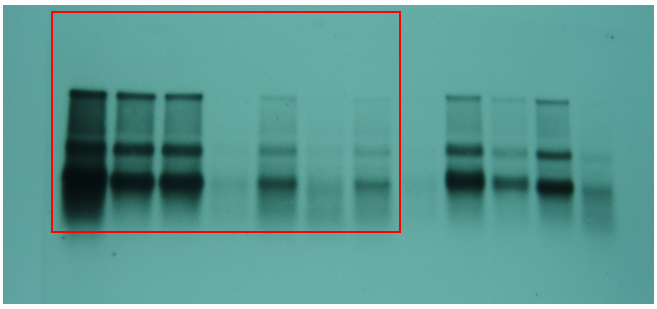

pep1 pep9 pep10

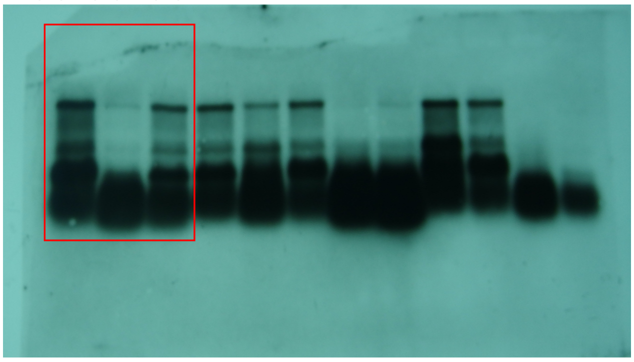

Northern blots showing the accumulation of PepMV gRNA and sgRNAs in *N. benthamiana* protoplasts after transfection with the indicated constructs.

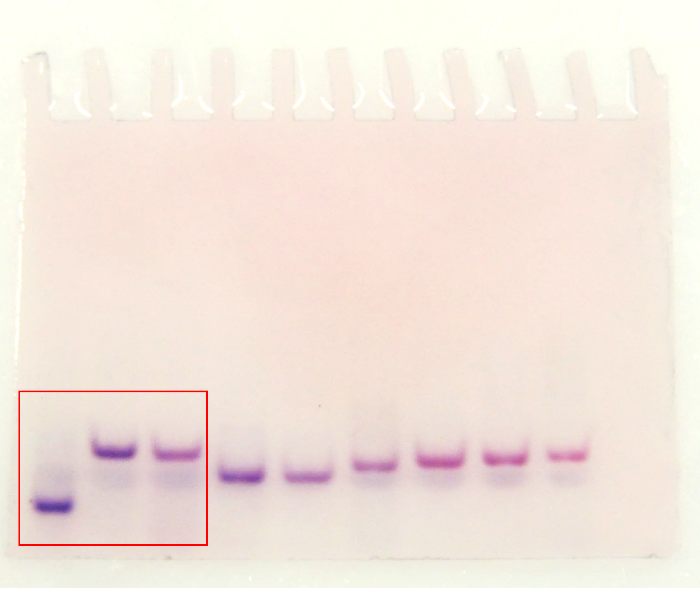

PepA PepB PepC

pH8

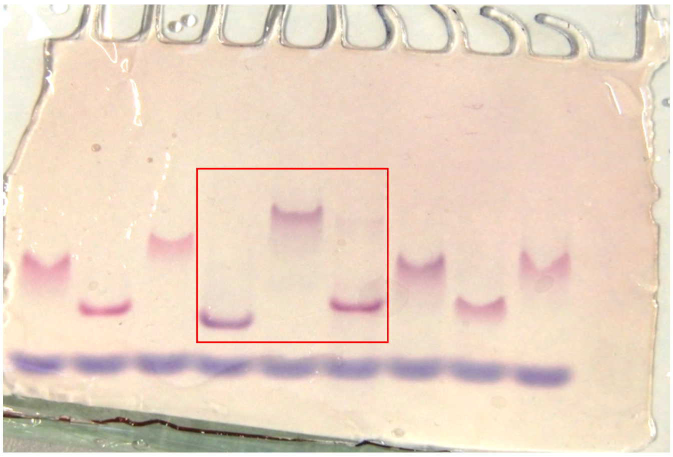

PepA PepB PepC

pH5

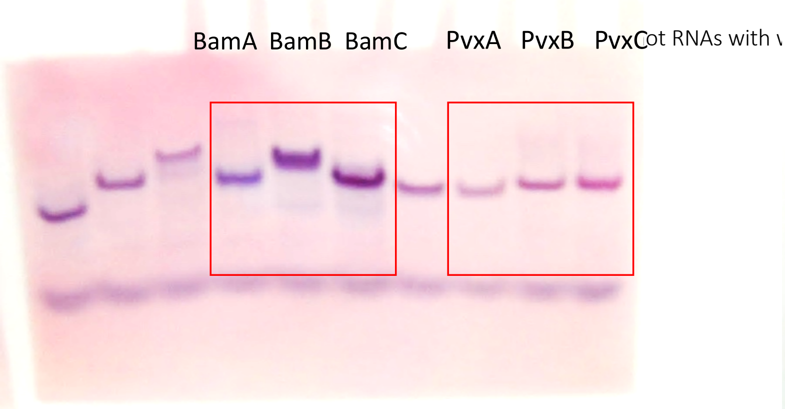

pH8

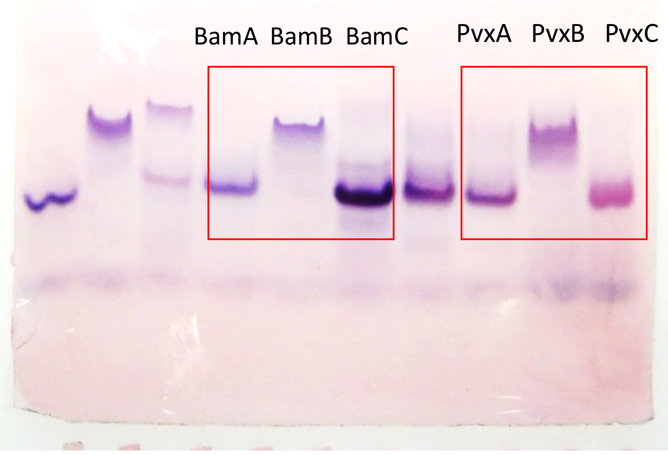

pH5

Native gel electrophoresis at pH 8 and 5 of PVX and BaMV pseudoknot RNAs. RNAs were visualized by Stains-All.

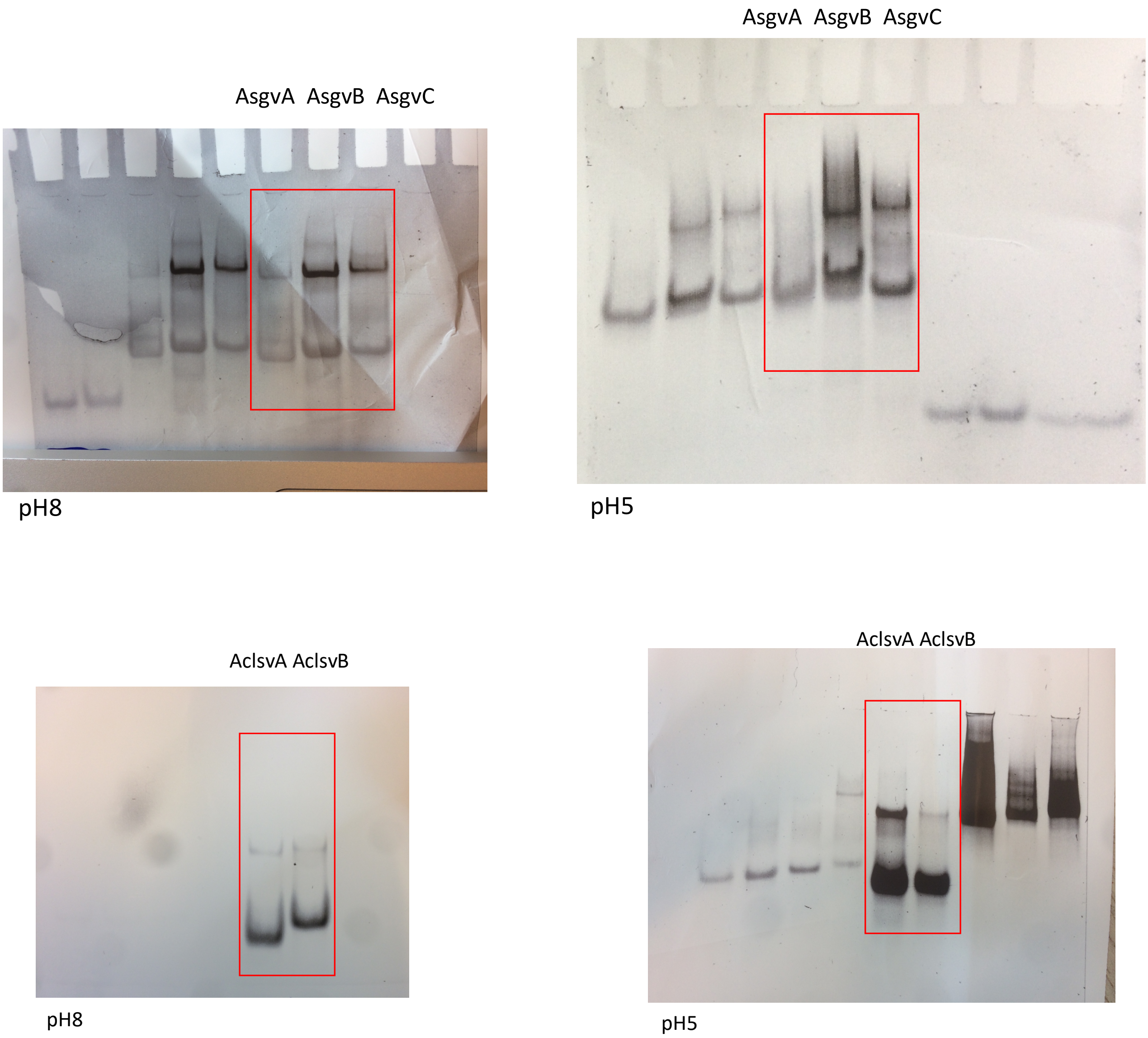

Native gel electrophoresis at pH 8 and 5 of **Apple stem grooving capillovirus (AsgvA, B, C) and** Apple chlorotic leaf spot trichovirus (AclsvA and B) pseudoknot RNAs. RNAs were visualized by EtBr staining.
